# Modeling the effects of steroid implant use on the environmental and economic sustainability of Brazilian beef production

**DOI:** 10.1093/tas/txab144

**Published:** 2021-09-20

**Authors:** Judith L Capper, Thiago B De Carvalho, Andrew S Hancock, Ocilon G Sá Filho, Isaac Odeyemi, David J Bartram

**Affiliations:** 1 Livestock Sustainability Consultancy, Harwell, Didcot, Oxfordshire, OX11 0HH, UK; 2 Unesp, Paulista State University, Universitaria Avenue, 3780 Botucatu, SP, Brazil; 3 Zoetis, Cherrywood Business Park , Loughlinstown, D18 K7W4, Co. Dublin, Ireland; 4 Zoetis, Rua Chucri Zaidan, 1240 Edifício Morumbi Corporate, Diamond Tower, São Paulo, Brazil

**Keywords:** beef, carbon footprint, economic viability, environmental impact, greenhouse gas, hormones

## Abstract

Brazilian beef systems contribute 14.9% of global beef production, therefore given climate change concerns, there is a clear need to reduce environmental impacts while maintaining economic viability. This study evaluated the hypothesis that steroid implant use in Brazilian beef cattle would reduce resource use, greenhouse gas (GHG) emissions and economic costs of production, thereby improving environmental and economic sustainability. A deterministic model based on beef cattle population demographics, nutrition and performance was used to quantify resource inputs and GHG emissions per 1.0 × 10^6^ kg of hot carcass weight (HCW) beef. System boundaries extended from cropping input manufacture to cattle arriving at the slaughterhouse. Beef systems were modeled using herd population dynamics, feed and performance data sourced from producers in four Brazilian states, with additional data from global databases. Implants were used in calves, growing and finishing cattle at low (LI), medium (MI), and high (HI) levels of performance enhancement, compared to nonimplanted (NI) controls. Feed use results were used in combination with producer-derived input costs to assess the economic impacts of implant use, including production costs and returns on investment. Improved FCE, ADG, and carcass weights conferred by implant use reduced the number of cattle and the time taken to produce 1.0 × 10^6^ kg HCW beef. Compared to NI controls, the quantities of feed, land, water and fossil fuels required to produce 1.0 × 10^6^ kg HCW beef was reduced in implanted cattle, with reductions proportional to the performance-enhancing effect of the implant (HI > MI > LI). Implant use reduced GHG emissions per 1.0 × 10^6^ kg HCW beef by 9.4% (LI), 12.6% (MI), or 15.8% (HI). Scaling up the MI effects to represent all eligible Brazilian cattle being implanted, revealed avoided GHG emissions equivalent to the annual exhaust emissions of 62.0 × 10^6^ cars. Economic impacts of implant use reflected the environmental results, resulting in a greater margin for the producers within each system (cow-calf through to finishing). The 6.13% increase in kg of HCW beef produced generates a cost reduction of 3.76% and an increase in the return on invested capital of 4.14% on average. Implants offer the opportunity for Brazilian beef producers to demonstrate their dedication to improving environmental and economic sustainability through improved productivity, although care must be taken to avoid negative trade-offs.

## INTRODUCTION

The sustainability of animal source foods (ASF, i.e., milk, meat and eggs) is an issue of considerable debate for all food stakeholders, from primary producers, processors, and retailers through to consumers, media, and government. The combination of an ever-expanding worldwide population (United Nations, 2019) and a rise in the global middle-class (Ponnampalam et al., 2019) is predicted to exponentially increase demand for ASF, yet this demand must be fulfilled in a sustainable manner. Sustainable ASF production systems balance environmental responsibility, economic viability and social acceptability, with each of these factors providing complementary and opposing forces at any point in time. The requirements for a sustainable system are therefore inherently plastic, which is evidenced by the fact that historical management systems and practices that were previously considered to be environmentally, economically or socially acceptable are now not viable. Future ASF systems will therefore need to consider innovative practices to allow a greater quantity of ASF to be produced using fewer resources (Capper, 2020) and at an affordable economic cost.

Considerable criticism is leveled at beef production systems for their contribution to environmental impacts, specifically the effect of greenhouse gas (GHG) emissions on climate change. According to the FAO (2013), livestock production accounts for 14.5% of global GHG emissions, with beef production contributing 41% of this total. The average global GHG emissions per kg of beef carcass weight (CW) are 47 kg CO_2_e, yet significant variation exists within this figure, with regional values ranging from 14 kg CO_2_e for Eastern European beef to 76 kg CO_2_e for beef produced in Southeast Asia (Opio et al., 2013). Given the differences in GHG emissions attributed to beef production from various systems and regions across the globe (Ogino et al., 2004; Casey and Holden, 2006; Pelletier et al., 2010; Capper, 2011, 2012; Cederberg et al., 2011; Capper and Hayes, 2012; Lupo et al., 2013; Nguyen et al., 2013; Rotz et al., 2013; Picasso et al., 2014; White and Capper, 2014; de Vries et al., 2015; Wiedemann et al., 2015; Legesse et al., 2016), it is therefore critical to examine the environmental impacts of regional beef systems in context, including related economic and social considerations, rather than applying blanket statistics.

Progress made in cattle genetics, nutrition, management and health over time has been key to reducing the environmental impacts of regional beef production systems (Alford et al., 2006; Capper, 2011; Wiedemann et al., 2015; Legesse et al., 2016) and this culture of continuous improvement must continue to ensure future sustainable cattle production. Improving productivity such that a specific quantity of beef may be produced using fewer animals or in less time has been shown to reduce GHG emissions and resource use in multiple studies (Beauchemin et al., 2011; Capper, 2011, 2012; Basarab et al., 2012; Lupo et al., 2013; Nguyen et al., 2013; White and Capper, 2014; Mogensen et al., 2015; Hyland et al., 2016; Murphy et al., 2017). Tools and technologies that allow producers to improve average daily gain (ADG), feed conversion efficiency (FCE), or slaughter weight will therefore become increasingly important in future beef production systems, providing that they are both economically viable and socially acceptable (Johnson et al., 2013; Capper, 2020).

Steroid hormone implants have been used in U.S. cattle production systems for decades, yet, to date, have not been registered in every country worldwide. The active hormones in implants (estrogens, androgens or their combination) increase muscle protein synthesis, reduce protein degradation, and improve cattle ADG (Parr et al., 2010; Beck et al., 2012, 2014; Strydom, 2016; Webb et al., 2017; Cleale et al., 2018); although these improvements are bound by the physical, metabolic and biochemical parameters that the animal is genetically programmed to achieve (Smith et al., 2020). Implant use within U.S. beef production systems demonstrably reduced the GHG emissions and resource use per kg of beef produced compared to controls in both live animal experiments (Basarab et al., 2012; Stackhouse-Lawson et al., 2012; Webb et al., 2017) and modeling exercises (Capper, 2011, 2012, 2013; White and Capper, 2014), yet, to date, the impacts of implant use in other regional beef systems have not been investigated in any significant detail. Productivity improvements conferred by implant use also improved economic returns in both feedlot and pasture-based steers according to Beck et al. (2014), and had positive economic impacts on beef production from heifers and bulls (Al-Husseini et al., 2014; Smith et al., 2020).

Compared to U.S. production, Brazilian systems tend to have lower productivity, with a lesser ADG, greater age at slaughter, and fewer cows weaning a live calf each year (Ferraz and Felício, 2010). These factors may be primarily attributed to the extensive, pasture-based nature of the majority of Brazilian production systems; seasonal differences in pasture growth between wet and dry seasons; and species-specific characteristics of *Bos indicus* cattle (Millen et al., 2011; Millen and Arrigoni, 2013). Mazzetto et al. (2015) demonstrated that improving productivity within characteristic Brazilian beef systems reduced GHG emissions, with similar conclusions reached by de Oliveira Silva et al. (2016), Cardoso et al. (2016) and Pashaei Kamali et al. (2016).

Given that Brazilian beef production contributes 14.9% of global beef (FAO, 2021), improving productivity within these systems would be predicted to have significant effects on total global resource use and GHG emissions. This study was designed to evaluate the hypothesis that steroid implant use within Brazilian beef production would reduce resource use, GHG emissions, and economic costs of production per unit of beef produced, thereby improving environmental and economic sustainability.

## MATERIALS AND METHODS

The environmental impacts (resource use, nutrient excretion and GHG emissions) of steroid implant use within Brazilian beef production were assessed using a deterministic Microsoft Excel-based model of cattle nutrition, metabolism and herd population parameters founded on life cycle assessment (LCA) principles. Economic costs of production were derived from feed use results produced by the environmental impact model, in combination with producer-derived input costs. This study used production data from commercial Brazilian beef operations collected via questionnaire, therefore approval from an animal care and use committee was not required.

The environmental model employed was based on the original models described by Capper (2011) and Capper (2012) with further modifications similar to those used in the dairy model described by Capper and Cady (2020). Beef production, including growing and harvesting crops for animal feed, were modeled according to practices and performance metrics typical of Brazilian beef production based on a case study with data collected by producer questionnaire. The case study, as defined by Yin (1994), was an empirical research activity that gathered material to examine a specific present-day event or action in a bounded environment. This allowed for intensive research on a specific case, identifying essential factors, processes, and relationships. Case studies were carried out via questionnaire in four Brazilian states—Goiás, Mato Grosso (MG), Mato Grosso do Sul (MGS), and São Paulo. According to the Brazilian Institute of Geography and Statistics (Instituto Brasileiro de Geografia e Estatística, 2021) the states of MG and Goiás account for approximately 14.9% and 10.6%, respectively, of the total cattle herd in Brazil, followed by the states of Minas Gerais (10.3%) and MGS (9.03%). Cattle in São Paulo represent 4.88% of the total. Data were collected from two cow-calf and two finishing operations in the states of MG and MGS, one bull feedlot in Goiás and a heifer feedlot in São Paulo. The latter two states were chosen because Brazil finished approximately 6.8 million animals in feedlot or semiconfined systems in 2019, with Goiás, São Paulo, MG, and MGS accounting for 73% of feedlot operations (USDA, 2019).

System boundaries extended from the production of feed and forage crops (including manufacture of cropping inputs, e.g., fertilizers and pesticides) through to and including transport of finished cattle to the slaughterhouse door. The impacts of postslaughter transportation, processing, packaging and consumption were not included; neither were specific on-farm technologies and practices (e.g., manure processing and application) not directly related to cattle feeding, management, and husbandry. A number of co- and byproducts originate from beef cattle production, including (but not limited to) leather, pharmaceuticals and bone meal. Ideally, system environmental impacts would be allocated between the principal product (beef) and all co- and byproducts, however, this was beyond the scope of the current investigation. To ensure that the results of the analysis were as conservative as possible, the decision was therefore made not to apply allocation within this analysis. The functional unit by which environmental impact was assessed was the production of 1.0 × 10^6^ kg (one million kg) of hot carcass weight beef (HCW).

The producer questionnaire collated data relating to cattle performance, feed, transportation, crop production and infrastructure, plus economic data relating to costs of production and revenues accrued. The baseline characteristics of these production systems are shown in Tables 1 and 2. In brief, the cow-calf operations contained mature cows and bulls, replacement heifers and bulls, and calves, grazing pasture (a 50:50 mixture of palisade grass—*Brachiaria brizantha*, and signal grass—*Brachiaria decumbens*) and supplemented with minerals. Nelore cows had a mature weight of 420 kg (MG) or 450 kg (MGS), milk yield, and composition according to Rodrigues Paulino et al. (2010) and a calving interval of 514 d or 479 d in MG and MGS, respectively. A combination of calving rate and calf mortality meant that 68.4% of cows weaned a live calf each year in MG and 72.7% of cows in MGS. Cow mortality rate, culling age, cow:bull ratio and stocking rate were similar in both cow-calf operations at 12.5%, 96 mo, 30:1 and 0.74 cows/ha, respectively. Replacement heifers calved for the first time at 42 mo and 300 kg (MG) or 38 mo and 320 kg (MGS). Mature Nelore bulls weighed an average of 800 kg (MG) or 780 (MGS) and were culled at 84 mo (MG) or 96 mo (MGS). Calves had a birthweight of 31 kg (MG) or 35 kg (MGS) and were weaned weighing 180 kg at an average of 240 d of age in both cow-calf systems. Weaned calves also grazed pasture but were also given supplemental urea, corn, and minerals (Table 1).

**Table 1.** Key data input metrics for Brazilian cow-calf operations

Data input	Mato Grosso	Mato Grosso do Sul
**Cows**		
Breed	Nelore	Nelore
Mature weight, kg	420	450
Pregnancy rate, %	73.2	85.6
Calving rate, %	71.3	76.5
Weaning rate, %	68.4	72.7
Calving interval, d	514	479
Cow:bull ratio	30:1	30:1
Age at culling, mo	96.0	96.0
Mortality/culling rate, %	12.5	12.5
Stocking rate, head/ha	0.74	0.74
Diet ingredients	Pasture, minerals	Pasture, minerals
**Replacement heifers**		
Age at first calving, mo	42	38
Weight at first calving, kg	300	320
Mortality rate, %	12.5	12.5
Stocking rate, head/ha	0.74	0.74
**Mature bulls**		
Breed	Nelore	Nelore
Mature weight, kg	800	780
Age at culling, mo	84	96
Mortality/culling rate, %	12.5	12.5
Stocking rate, head/ha	0.74	0.74
**Preweaned calves**		
Calf birthweight, kg	31	35
Newborn mortality rate, %	4	5
Preweaned calf mortality, %	2	1
Age at weaning, d	240	240
Weight at weaning, kg	180	180
Stocking rate, head/ha	0.74	0.74
**Weaned calves**		
Mortality rate, %	1.4	2.5
Stocking rate, head/ha	0.74	0.74
Diet ingredients	Pasture, urea, corn, minerals	Pasture, urea, corn, minerals
**Infrastructure**		
Manure management	Spread on fields	Spread on fields
Truck fuel efficiency (km/liter)	2.5	2.5
Transport distance for bought-in feed, km	300	140
Transport distance for bought-in fertilizer, km	300	280

**Table 2.** Key data input metrics for Brazilian finishing cattle operations

	Mato Grosso	Mato Grosso do Sul	Goiás	São Paulo
Data input	Finishing farm	Finishing farm	Bull feedlot	Heifer feedlot
Mortality rate, %	1.4	2.5	1.0	0.3
Age at slaughter, mo	36.0	36.0	27.3	26
Weight at slaughter, kg	550	509	525	487
Dressing percentage, %	53.0	53.0	56.0	52.0
Stocking rate, head/ha	0.95	0.95	–	–
Diet ingredients	Pasture, urea, corn, minerals	Pasture, urea, corn, minerals	Corn silage, corn, soybean meal, cottonseed meal, limestone, urea	Sugarcane silage, corn silage, corn, cottonseed meal, citrus pulp, urea
Manure management	Spread on fields	Spread on fields	Spread on fields/lagoon storage	Spread on fields/lagoon storage
Transport distance to finishing farm or feedlot, km	150	200	200	584
Truck capacity, head	40	40	42	42
Transport distance to slaughterhouse, km	30	80	20	135
Truck capacity, head	27	27	27	27
Truck fuel efficiency (km/L)	2.5	2.5	2.5	2.5
Transport distance for bought-in feed, km	300	250	25	500
Transport distance for bought-in fertilizer, km	300	180	25	500

The majority of bull calves were grown and finished for beef, whereas, due to the relatively late age at first calving, a higher proportion of heifers were retained as herd replacements. Weaned calves destined for beef were either grown and finished on pasture on a finishing farm (90% of calves) characteristic of a significant proportion of Brazilian beef cattle (Ferraz and Felício, 2010), or grown on pasture to 24 mo of age (Nelore bulls and heifers) or 20 mo (crossbred heifers) before being finished in a feedlot (10% of calves). A small proportion (1.7% of growing cattle destined for beef) of crossbred feedlot heifers originated from dairy production. Although resource use and GHG emissions invested in production of these dairy calves would ideally have been allocated for, this was outside the scope of the current study, especially given the minor contribution of these cattle to total beef production. Mortality rates in the finishing farm and feedlot were low, ranging from 0.3% to 2.5% as shown in Table 2. Liveweight (LW), age and ADG for growing cattle originating in the MG or MGS cow-calf operations are shown in Table 3. The age at slaughter varied from 26 mo (heifer feedlot) to 36 mo (both finishing farms), with slaughter weights ranging from 487 kg (heifer feedlot) to 550 kg (MG finishing farm). Dressing percentages averaged 53.0% for finishing farm cattle, 56.0% for feedlot bulls and 52.0% for feedlot heifers. Finishing farm diet ingredients were based on pasture (same species as cow-calf operations), urea, corn, and minerals, depending on age and production stage; whereas bull feedlot diets contained corn silage, corn, soybean meal, cottonseed, limestone and urea; and heifer feedlot diets comprised sugarcane silage, corn silage, corn, cottonseed meal, citrus pulp, and urea.

**Table 3.** Baseline performance data^*a*^ for growing cattle within the model

Cattle group	Mato Grosso				Mato Grosso do Sul			
	Mean age, mo	Start weight, kg	End weight, kg	ADG, kg/d	Mean age, mo	Start weight, kg	End weight, kg	ADG, kg/d
**Preweaned calves**								
Nelore bull	3.9	31	180	0.621	3.9	35	180	0.604
Nelore heifer	3.9	31	180	0.621	3.9	35	180	0.604
Crossbred heifer	3.9	39	190	0.631	3.9	39	190	0.631
**Growing cattle destined for finishing farm**								
Nelore bull	16.0	180	402	0.454	16.0	180	380	0.408
Nelore heifer	16.0	180	372	0.392	16.0	180	348	0.342
**Cattle in finishing farm**								
Nelore bull	30.0	402	568	0.454	30.0	380	529	0.408
Nelore heifer	30.0	372	515	0.392	30.0	348	472	0.342
**Growing cattle destined for feedlot**								
Nelore bull	16.0	180	360	0.367	16.0	180	360	0.367
Nelore heifer	16.0	180	308	0.260	16.0	180	308	0.260
Crossbred heifer	14.0	190	346	0.424	14.0	190	346	0.424
**Finishing cattle in feedlot** ^*b*^								
Nelore bull	25.7	360	526	1.650	25.7	360	526	1.650
Nelore heifer	26.0	308	456	1.222	26.0	308	456	1.222
Crossbred heifer	22.0	346	519	1.417	22.0	346	519	1.417

^*a*^All performance data were either supplied directly from the beef operations involved in the study (ages and weights at start and end of each production stage) or derived from the modeling analysis (interim ages and weights, ADG, DMI).

^*b*^Cow-calf and finishing operations were based in Mato Grosso and Mato Grosso do Sul; bull feedlot was based in Goiás and heifer feedlot was based in São Paulo.

Within each regional (MG or MGS) simulation, there existed 30 groups of cattle: one group of lactating cows; one group of dry cows; two groups of barren cows; one group of mature bulls; four groups of replacement heifers (weaning—12 mo, 12–24 mo, 24 mo—conception, conception-calving); three groups of replacement bulls (weaning—12 mo, 12–24 mo, 24–36 mo); three groups of preweaned calves (Nelore bulls, Nelore heifers, crossbred heifers); six groups of growing cattle destined for the feedlot (Nelore bulls, Nelore heifers, crossbred heifers at both weaning—12 mo and 12 mo-feedlot entry); three groups of feedlot cattle (Nelore bulls, Nelore heifers and crossbred heifers); and six groups of finishing farm cattle (Nelore bulls and Nelore heifers, at weaning—12 mo, 12–24 mo, and 24–36 mo). Agricultural Modeling and Training Systems (AMTS, 2018) ration formulation software was used to formulate balanced, nutritionally-appropriate rations for cattle within each animal group according to LW, production level (pregnancy, lactation and/or growth) and the diet ingredients data supplied by the Brazilian cattle operations (Tables 1 and 2). The same software was used to predict daily dry matter intake (DMI), nutrient requirements, voluntary water intake, manure output, and enteric methane (CH_4_) emissions. The fraction of nitrogen emitted as enteric nitrous oxide was calculated from data reported by Kaspar and Tiedje (1981) and Kirchgessner et al. (1991). Diet formulation for each animal group allowed quantification of the population nutrient requirements and therefore the cattle (feedstuffs, water) and crop (fertilizer, pesticides, fuels) inputs associated with beef production.

Implants were assumed to be used in all eligible cattle destined for beef production, excluding those reared as heifer or bull replacements for the cow-calf operation, i.e., preweaned calves, growing calves and finishing cattle. The aim of this paper was to investigate the environmental and economic impacts of generic implant use on the Brazilian beef system, rather than examining the effects of specific types (e.g., estrogen vs. trenbolone acetate) or commercial brands of implant; or the impacts on specific breeds or groups of cattle. Changes in ADG, FCE, CW, and dressing % conferred by implant use were derived from those published by Duckett et al. (1996) at three levels of performance enhancement—low (LI), medium (MI), and high (HI), as shown in Table 4. The control scenario (no implants) was designated “NI”. Diet formulation and DMI were adjusted according to implant use, via AMTS (2018) software.

**Table 4.** Effects of steroid implants at three levels of performance enhancement on beef cattle key performance indicators, derived from Duckett et al. (1996)

Key performance indicator	Level of performance enhancement		
	Low	Medium	High
ADG	+12.6%	+18.0%	+23.4%
FCE	+5.60%	+8.00%	+10.4%
Slaughter weight	+1.75%	+2.50%	+3.25%
Dressing %	+1.75%	+2.50%	+3.25%
Carcass weight^*a*^	+3.5%	+5.00%	+6.50%

^*a*^A function of the combined improvements in slaughter weight and dressing percentage.

Manure in grazing operations (cow-calf and finishing farms) deposited onto pasture, whereas feedlot operations employed a combination of spreading on fields and lagoon storage (Tables 1 and 2). Methane emissions from manure were estimated using methodology prescribed by the Intergovernmental Panel on Climate Change (IPCC, 2019) based on the quantity of volatile solids excreted, maximum methane-producing potential and conversion factors for both grazing and liquid slurry storage systems in the tropical moist climate characteristic of Brazil. Intergovernmental Panel on Climate Change (IPCC, 2006) emission factors were used to calculate nitrous oxide (N_2_O) emissions from manure stored as slurry, and the nitrogen losses from manure deposited on soil according to manure nitrogen content and the emissions factors from Lesschen et al. (2011).

Cropping yields were supplied from the commercial beef operations participating in the study (corn silage) or derived from FAOSTAT (FAO, 2021) based on 5-yr averages of Brazilian cropping yields (all other crops), as shown in Table 5. Fertilizer data for individual crops were sourced from FAO (2006); pesticide data from Pignati et al. (2014), Pollak (2020) and Coltro et al. (2009); and diesel use from Camargo et al. (2013), Pryor et al. (2017), Cotton Inc. (2012), and Franco Junior et al. (2014). If co- or byproduct feeds resulted from a specific crop (e.g., soybean meal, cottonseed meal, or citrus pulp), the yields and resource inputs required to produce the feed were pro-rated according to the proportional mass of the co- or byproduct compared to the main product. Inorganic diet ingredients (minerals, limestone, urea) had no land, fertilizer or pesticide footprint and were consumed in such low quantities as to be considered de minimis. According to the results of the Brazilian 2017 Census of Agriculture (Instituto Brasileiro de Geografia e Estatistica, 2021), less than 2% of land in MG, MGS, or Goiás was irrigated, only 6.83% of land in São Paulo, and over 50% of the irrigated area was used for rice and sugarcane (Flach et al., 2020). Irrigation water for feed crop or pasture production was therefore not considered to represent a significant resource use and was not included in the analysis.

**Table 5.** Yields and cropping input data for Brazilian feed crop production

Crop	Yield, kg/ha	Resource use, kg/ha				Diesel, l/ha
		N	P	K	Pesticides	
Pasture^*a*^	–	–	–	–	–	–
Corn silage^*b*^	50.0 × 10^3^	75.6	56.0	56.0	3.25	111.5
Sugarcane silage^*c*^	84.4 × 10^3^	55.0	51.0	110.0	2.60	63.0
Corn grain^*d*^	5.14 × 10^3^	40.0	35.0	33.0	3.25	97.6
Soybean meal^*e*^	2.44 × 10^3^	8.0	66.0	62.0	5.09	32.8
Cottonseed meal^*f*^	3.19 × 10^3^	83.0	130.0	122.0	10.59	40.9
Citrus pulp^*g*^	15.9 × 10^3^	55.0	24.0	45.0	25.62	229.4

^*a*^50:50 mixture of palisade grass (*Brachiaria brizantha*) and signal grass (*Brachiaria decumbens*). Data from the cow-calf, finishing farm and feedlots revealed no inorganic fertilizer or pesticide inputs.

^*b*^Corn silage yield data supplied from the farms involved in the study; fertilizer data from FAO (2006); pesticide data from Pignati et al. (2014) calculated using soybeans as a reference point; diesel use according to Camargo et al. (2013).

^*c*^Sugarcane silage yield data from the FAOSTAT database (FAO, 2021) specific to Brazilian production and averaged over five cropping years (2014–2019) and pro-rated for a relative freshweight silage yield factor of 2.2 (Waclawovsky et al., 2010); fertilizer data from FAO (2006); pesticide data from Pignati et al. (2014) calculated using soybeans as a reference point; diesel use calculated from data published by Pryor et al. (2017).

^*d*^Corn grain yield data from the FAOSTAT database (FAO, 2021) specific to Brazilian production and averaged over five cropping years (2014–2019); fertilizer data from FAO (2006); pesticide data from Pignati et al. (2014) calculated using soybeans as a reference point; diesel use according to Camargo et al. (2013).

^*e*^Soybean meal yield data from the FAOSTAT database (FAO, 2021) specific to Brazilian production, averaged over five cropping years (2014–2019), and pro-rated for a relative meal yield of 78.3% (North Carolina Soybean Producers Association Inc., 2014); fertilizer data from FAO (2006); pesticide data from Pollak (2020); diesel use according to Camargo et al. (2013).

^*f*^Cottonseed meal yield data from the FAOSTAT database (FAO, 2021) specific to Brazilian production, averaged over five cropping years (2014–2019), and pro-rated for a relative meal yield of 81.5% (Sawan, 2016); fertilizer data from FAO (2006); pesticide data from Pignati et al. (2014) calculated using soybeans as a reference point; diesel use according to Cotton Inc (2012).

^*g*^Citrus pulp yield data from the FAOSTAT database (FAO, 2021) specific to Brazilian production, averaged over five cropping years (2014–2019), and pro-rated for a relative pulp yield of 60.0% (Feedipedia, 2021); fertilizer data from FAO (2006); pesticide data from Coltro et al. (2009); diesel use calculated from data published by Franco Junior et al. (2014).

With the exception of pasture and corn silage, all feeds were grown off-farm. Feed, fertilizer and pesticide transport was accounted for based on distance data (ranging from 25 to 500 km, Tables 1 and 2) and a fuel usage efficiency of 2.5 km/L supplied by the commercial beef operations involved, a truck capacity of 37,000 kg (Fliehr, 2013); and a diesel energy content of 34.8 MJ/L. The aforementioned fuel efficiency data, in conjunction with data supplied by the commercial beef operations on the number of head of cattle carried per truck (27–42 head, depending on LW), the transport distances between production phases (e.g., cow-calf to finishing farm or feedlot) and transport distances to the slaughterhouse allowed fossil fuel use and associated GHG emissions from cattle transport to be quantified.

Carbon dioxide emissions from fertilizer and pesticide manufacture were derived from West and Marland (2002). Due to a lack of reliable data and the number of assumptions involved in applying a land use factor to crop and pastureland, carbon sequestered into soil was not included in the model calculations. Total GHG emissions were calculated by applying carbon dioxide-equivalent 100-yr factors from IPCC (2013) to CH_4_ and N_2_O to calculate the total carbon footprint as the sum of all CH_4_, N_2_O, and CO_2_ emissions expressed in CO_2_e, per functional unit (1.0 × 10^6^ kg of HCW beef).

The savings in water use, energy use and GHG emissions conferred by implant use at the average level of performance enhancement were converted into consumer-friendly metrics in terms of mean annual human use per capita (water and energy), car-equivalents (GHG emissions) and tree-equivalents (GHG emissions). Water savings were transformed into the mean annual usage per capita in Brazil, using data from Marli (2018) which cited an individual usage of 108.4 L per capita per day (39,566 L per year). In terms of energy savings conferred by implant use, the difference in energy use between “no implant” and “average performance enhancement” was converted into multiples of Brazilian per capita consumption using the figure of 53 kWh per month (636 kWh annually) published by Maçeira et al. (2017).

The car equivalents (annual kg of GHG expressed as CO_2_e per car) were based on the average distance travelled for passenger cars per year in Brazil (13,797 km), a fuel efficiency of 18.2 km/L, and GHG emissions for Brazilian fuel of 1.22 kg CO_2_/L based on 57% ethanol and 43% diesel (Glensor and Muñoz, 2019). Tree equivalents (annual CO_2_ sequestered by a mature tree per year) were based on the geospatial data published by Domke et al. (2020) which reported a mean annual CO_2_ sequestration rate of 5.06 kg CO_2_ per mature tree. These equivalents were scaled to up to represent the impacts if all Brazilian growing and finishing cattle were implanted, based on a total Brazilian beef production of 10.2 × 10^6^ metric tonnes in 2019 (FAO, 2021), excluding beef produced from cull cattle.

Feed use data outputs from the environmental model were used in conjunction with performance data relating to ADG, FCE, and CW and economic data from the producer questionnaires (Table 6 and the “no implant” section of Table 9) to assess the economic impacts of implant use. The financial cost of using the implant was based upon a 5.0% increase on the 2020 U.S. recommended retail price for Synovex, Synovex One Grass and Synovex Plus (all produced by Zoetis, Parsippany NJ, USA), as shown in Table 6, with an additional 0.017 h labor cost factored in per implant for the implantation process. All economic input costs were converted from Brazilian real to USD ($) based upon the average conversion rate for 2020, equal to 5.16 reals per USD.

**Table 6.** Economic costs of steroid implants used in Brazilian beef cattle

Animal	Number of implants	Implant cost per animal^*a*^
Calf	1 (3 mo of age)	$1.16
	1 (6 mo of age)	$1.16
Growing/finishing bull (180–360 kg)	1 (15 mo of age)	$1.16
	1 (24 mo of age)	$4.46
Growing/ finishing heifer (180–360 kg)	1	$4.46
Feedlot finisher	1	$2.91

^*a*^Based upon a 5.0% increase on the 2020 U.S. recommended retail price for Synovex C^®^, Synovex One Grass^®^ and Synovex Plus^®^ (all produced by Zoetis, Parsippany NJ, USA), plus 0.017 h labor cost per implant for the implantation process.

The economic assessment was founded upon the evaluation structures proposed by Matsunaga et al. (1976), including the following components:

- Revenue (R) is equal to sales of cattle and by-products from the operation
- Effective operating cost (COE) comprises all items considered to be variable costs or direct expenses represented by the cash disbursement recorded throughout the production cycle, being a function of the quantity used and the economic value per unit
- Total operating cost (COT) refers to the portion of indirect costs represented by linear depreciation, provision of labor and fees associated with the production process and family labor.

After determining revenues and costs, the gross margin (revenue - effective operating cost) and net margin (revenue – total operating cost) were calculated, in addition to the return on investment (ROI) of implant use. The ROI acted to quantify the efficiency of an investment or to evaluate the efficiency of a series of different investments, i.e., the capital gained or lost through implant use compared to the amount of capital invested.

## RESULTS AND DISCUSSION

Livestock industries across the globe are under increasing pressure to improve all three facets of sustainability: environmental responsibility, economic viability and social acceptability. As such, the United Nations (2015) collated a list of sustainable development indicators ranging from zero hunger to climate action that may be used as the foundation by which to assess the sustainability of systems, products or services. The ultimate goal is to supply the entire global population with sufficient affordable, nutritious food for optimal human health and development, while reducing environmental impacts and balancing an equitable livelihood for producers. As discussed by Eisler et al. (2014), the obvious constraints conferred by a finite land base mean that improving livestock productivity will be key to increasing the output of ASF per hectare of land, although the mechanisms and practices to achieve this in differing regions and systems will vary according to economic, environmental, cultural and practical considerations.

The role of improved cattle performance in reducing environmental impacts from beef production via the “dilution of maintenance” effect was discussed by Capper (2011) with reference to historical vs. modern beef production; and again in terms of extensive vs. intensive systems (Capper, 2012). To summarize the concept: every animal has a daily maintenance nutrient requirement which must be met before further nutrients may be partitioned into pregnancy, lactation or growth. If beef cattle productivity (ADG) improves, the total daily nutrient requirement increases, but the proportion of nutrients apportioned to maintenance are diluted out over a greater number of production units (kg of LW gain) and lesser amount of time. This effect may be enhanced by the use of feed additives and other technologies that improve the efficiency of converting forages and concentrates into beef. Furthermore, some specific implants may play a particular role here in enhancing the conversion of low-quality native forages into meat. For example, Paisley et al. (1999) reported that steers grazing dormant range grasses had low ADG, yet trenbolone acetate implants were effective in increasing weights gains compared to controls and to maintaining these weight gains through subsequent grazing and finishing periods. This would be particularly advantageous during the dry season in Brazilian grazing systems.

At the herd level, if slaughter weight is improved, the population maintenance requirement is spread over a greater quantity of beef produced, therefore it is possible to produce more beef from the same quantity of cattle or maintain production of a set quantity of beef using fewer total cattle (both growing/finishing cattle and supporting population). As shown in Table 7, using implants reduced age at slaughter and increased both slaughter weight and CW, which reduced the numbers of cattle required to produce 1.0 × 10^6^ kg of HCW beef from 2.55 × 10^4^ total cattle (NI) to a minimum of 2.40 × 10^4^ head (HI), a 5.88% decrease (Table 8).

**Table 7.** Productivity gains conferred by implant use within Brazilian beef production at low, medium or high levels of performance enhancement compared to a control (no implants) scenario

Finishing system	Cattle group	Productivity metric	Level of implant performance enhancement			
			No implants (NI)	Low (LI)	Medium (MI)	High (HI)
Finishing farm	Nelore heifers	Slaughter age, mo	36.0	32.0	30.6	29.4
		Slaughter weight, kg	515	524	528	532
		Carcass weight, kg	273	283	287	291
	Nelore bulls	Slaughter age, mo	36.0	32.2	30.8	29.6
		Slaughter weight, kg	568	578	582	586
		Carcass weight, kg	301	312	316	321
Feedlot	Nelore heifers	Slaughter age, mo	28.0	26.9	26.5	26.1
		Slaughter weight, kg	456	464	468	471
		Carcass weight, kg	242	250	254	258
	Crossbred heifers	Slaughter age, mo	24.0	22.9	22.5	22.2
		Slaughter weight, kg	519	528	532	535
		Carcass weight, kg	275	285	289	293
	Nelore bulls	Slaughter age, mo	27.3	26.3	26.0	25.7
		Slaughter weight, kg	526	535	539	543
		Carcass weight, kg	279	288	293	297

**Table 8.** Resource use and greenhouse gas emissions associated with producing 1 × 10^6^ kg of HCW beef from baseline Brazilian beef systems (no implants) or with steroid hormone implants at low, medium and high levels of performance enhancement

	No implants (NI)	Performance enhancement with implants		
		Low (LI)	Medium (MI)	High (HI)
**Animals** ^*a*^				
Total breeding cattle, head	6.49 × 10^3^	6.28 × 10^3^	6.20 × 10^3^	6.12 × 10^3^
Total preweaned calves, head	4.57 × 10^3^	4.43 × 10^3^	4.37 × 10^3^	4.31 × 10^3^
Total replacement heifers, head	3.41 × 10^3^	3.31 × 10^3^	3.26 × 10^3^	3.22 × 10^3^
Total breeding bulls, head	0.936 × 10^3^	0.906 × 10^3^	0.894 × 10^3^	0.882 × 10^3^
Total feedlot cattle, head	1.01 × 10^3^	0.979 × 10^3^	0.966 × 10^3^	0.954 × 10^3^
Total finishing farm cattle, head	9.07 × 10^3^	8.79 × 10^3^	8.67 × 10^3^	8.55 × 10^3^
Total cattle, head	2.55 × 10^4^	2.47 × 10^4^	2.44 × 10^4^	2.40 × 10^4^
**Resource use**				
Feedstuffs^*b*^, kg	1.96 × 10^8^	1.81 × 10^8^	1.75 × 10^8^	1.70 × 10^8^
Drinking water, L	1.27 × 10^8^	1.16 × 10^8^	1.12 × 10^8^	1.08 × 10^8^
Land, ha	2.19 × 10^4^	2.02 × 10^4^	1.95 × 10^4^	1.89 × 10^4^
N fertilizer, kg	10.9 × 10^3^	9.40 × 10^3^	8.82 × 10^3^	8.26 × 10^3^
P fertilizer, kg	9.68 × 10^3^	8.33 × 10^3^	7.81 × 10^3^	7.31 × 10^3^
K fertilizer, kg	9.21 × 10^3^	7.92 × 10^3^	7.42 × 10^3^	6.94 × 10^3^
Pesticides, kg	6.14 × 10^4^	5.67 × 10^4^	5.48 × 10^4^	5.30 × 10^4^
Fossil fuels, MJ	10.4 × 10^5^	9.04 × 10^5^	8.53 × 10^5^	8.03 × 10^5^
**Waste outputs**				
N excretion, kg	5.55 × 10^5^	4.97 × 10^5^	4.76 × 10^5^	4.57 × 10^5^
P excretion, kg	5.33 × 10^4^	4.77 × 10^4^	4.57 × 10^4^	4.38 × 10^4^
Manure, kg	1.63 × 10^8^	1.47 × 10^8^	1.41 × 10^8^	1.35 × 10^8^
**Gas emissions**				
Methane, kg	1.22 × 10^6^	1.11 × 10^6^	1.07 × 10^6^	1.03 × 10^6^
Nitrous oxide, kg	8.73 × 10^3^	7.82 × 10^3^	7.50 × 10^3^	7.20 × 10^3^
Greenhouse gases from livestock, kg CO_2_e	4.42 × 10^7^	4.01 × 10^7^	3.87 × 10^7^	3.73 × 10^7^
Greenhouse gases from cropping, kg CO_2_e	1.24 × 10^6^	1.24 × 10^6^	1.24 × 10^6^	1.24 × 10^6^
Greenhouse gases from manure application, kg CO_2_e	1.35 × 10^6^	1.21 × 10^6^	1.16 × 10^6^	1.11 × 10^6^
Greenhouse gases from transport, kg CO_2_e	5.65 × 10^4^	5.18 × 10^4^	5.00 × 10^4^	4.83 × 10^4^
Total greenhouse gases, kg CO_2_e	4.69 × 10^7^	4.25 × 10^7^	4.10 × 10^7^	3.95 × 10^7^
Total greenhouse gases kg CO_2_e per kg HCW beef	46.9	42.5	41.0	39.5

^*a*^Actual cattle numbers (head), not pro-rated for time spent in the system.

^*b*^Freshweight.

The improvements in beef cattle productivity conferred by implant use are well documented and are summarized in the reviews by Duckett and Pratt (2014), Smith and Johnson (2020), Aboagye et al. (2021) and Aroeira et al. (2021). The combination of improved slaughter weight, FCE and ADG in implanted cattle conferred considerable reductions in renewable resource use per 1.0 × 10^6^ kg beef, with up to 0.26 × 108 kg less feed (a 13.3% reduction) and 0.30 × 10^4^ ha less land (a 13.7% reduction) required to maintain production in the HI scenario compared to the control (NI). These results reflect the extensive nature of Brazilian beef production in that pastureland represented 98.5% of the total land savings, with very little supplemental feed across the entire nonfeedlot population, and feedlot cattle only being fed for an average of 3.8 mo in the NI scenario. Similarly, Pashaei Kamali et al. (2016) reported that the concentrate feed required by incorporating a 120 d feedlot finishing period into Brazilian beef operations only accounted for 2.1% of total land use per unit of beef. Land use per kg of beef HCW within the current study is within the ranges previously reported for Brazilian systems (Cederberg et al., 2009; Dick et al., 2015; Pashaei Kamali et al., 2016), yet it should be noted that the current study did not include an allotment for deforestation, as all pasture and cropland within the analysis was assumed to have been deforested some years previously. Nevertheless, conversion of Amazon or Cerrado rainforest into pastureland for cattle production is a highly controversial issue that must be addressed in any forum discussing Brazilian beef production. Zu Ermgassen et al. (2020) ascribed 41% of deforestation in the Amazon to cattle operations, excluding land which was then used for soybean production, and noted that although many processing companies had zero deforestation policies in place, these were not always upheld. The deforestation/cattle issue is not as simple as it often appears, however—as discussed by Fearnside (2005), under Brazilian law, land ownership may be demonstrated by “improving” the land such that rainforest is felled and a crop or livestock placed upon the land. Pastureland and cattle are simply the objects by which land ownership is established and maintained. It could therefore be suggested that if sugarcane, quinoa or avocados could be grown upon deforested land more easily than cattle, these crops might be subject to the same deforestation-related opposition as cattle operations. Nevertheless, deforestation is a significant environmental and social issue—its importance must not be underestimated.

Cerri et al. (2018) concluded that improving Brazilian cattle operation productivity in the Cerrado region would be an effective mechanism by which to mitigate Amazon deforestation, with similar conclusions reached by Latawiec et al. (2014), de Oliveira Silva et al. (2015) and Oliveira et al. (2020). Indeed, Martha et al. (2012) reported that cattle productivity improvements between 1950 and 2006 saved 525 million ha of Brazilian land, an area 25% greater than the Amazon biome. The low stocking rates characteristic of Brazilian production are not sustainable in the long term—land use per unit of beef could be reduced further by implementing best management practices (Pashaei Kamali et al., 2016), improved pasture cultivars (Jank et al., 2014) or feedlot finishing (Vale et al., 2019). These opportunities are limited by Brazilian climatic seasonality, however, Cardoso et al. (2016) modeled a variety of beef intensification scenarios and concluded that improving pasture quality, adding a 75 d feedlot finishing period and improving reproductive efficiency would confer up to a seven-fold reduction in land use and cut GHG emissions per kg CW by 49.6%, even when N fertilizer use was accounted for.

The concept of improved productivity reducing deforestation through land sparing was expanded upon by Cohn et al. (2014), who quantified the impacts of either taxing conventional cattle production or subsidizing semi-intensive cattle production (in which pasture productivity was assumed to double), revealing that either initiative would considerably reduce GHG emissions even if decoupled from direct actions to reduce deforestation. Stabile et al. (2020) proposed a four-pronged approach to reducing deforestation: eliminating land grabbing and speculation; eliminating deforestation on private lands; incentivizing improved productivity on medium and large ranches; and providing technical assistance and education to smallholder farmers such that sustainability could be improved. The latter two initiatives fit within the context of this paper in terms of implementing production-enhancing technologies (PET) that have already been adopted elsewhere to improving total beef production and reduce land use. For example, increasing annual beef output per land unit area by 150% (from 60 kg/ha to 150 kg/ha) on 21% of existing Brazilian rangeland would free enough land to meet production targets and expand crop production, without increasing deforestation (Stabile et al., 2020).

**Figure 1.**
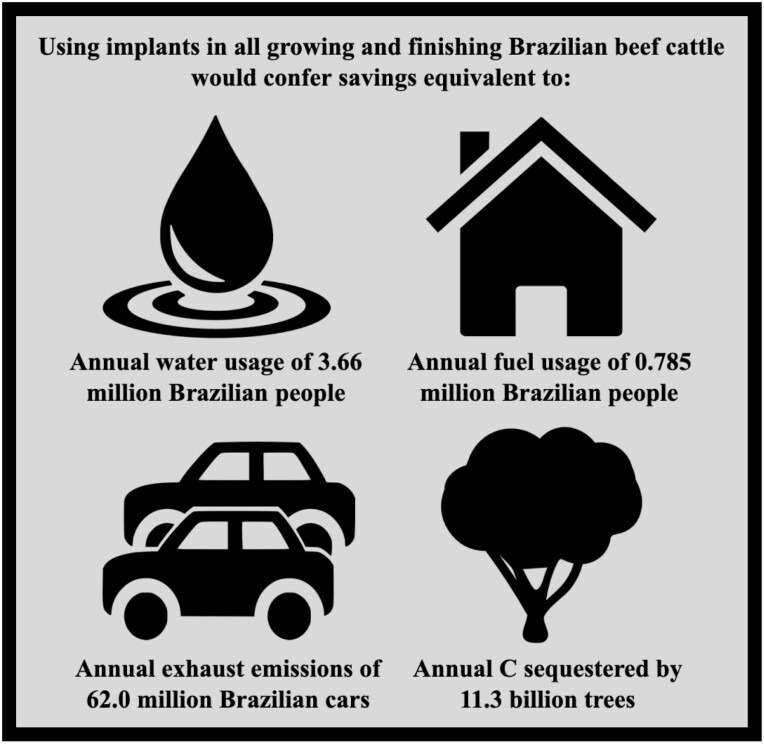
Water, fossil fuel and GHG emissions savings conferred by implant use in all growing and finishing Brazilian beef cattle, expressed in terms of annual usage.

Water use within livestock production is a significant global concern as it is the first limiting resource for many agricultural products and may be significantly impacted by deforestation in tropical and subtropical regions. Any technologies or practices that would allow water to be spared while maintaining beef production would therefore be environmentally favorable. The quantity of water attributed to producing a unit of beef summarized by Doreau (2012) ranged from 3 to 540 L of water/kg, varying by system, region and methodology. Given this considerable variation, it is not altogether surprising that the water use within the current study, which ranged from 1.08 × 10^8^ l per 1.0 × 10^6^ kg HCW beef (HI) to 1.27 × 10^8^ L per 1.0 × 10^6^ kg HCW beef (NI), are within the cited limits, although these quantities are considerably lower than those cited by Lathuillière et al. (2019) for beef production systems in MG State. The relatively low water use means that the saving of 0.15 × 10^8^ l conferred by implant use with a medium performance impact (scenario MI) is equal to the annual usage of only 377 Brazilian people, yet if this is scaled-up to represent all eligible Brazilian beef cattle being implanted, would be sufficient to supply 3.66 × 10^6^ people with their annual water needs (Figure 1). Recent studies have demonstrated that water intake is significantly lower in *Bos indicus* than *Bos taurus* cattle, which might confer greater advantage to Brazil in terms of producing more beef with less water utilization, compared to other regions (Valente et al., 2015, Cappellozza et al., 2020).

It should be noted however, that the lower water requirement per unit of HCW beef in the current study is not necessarily directly comparable to other studies within the region (Lathuillière et al., 2019), because although the environmental model allowed irrigation water to be quantified, it was not a component of total water use on the beef operations with the study. In isolation, results from the current study would therefore imply an environmental advantage of Brazilian production in the global context, nonetheless, it would be interesting to be able to evaluate the environmental benefits and trade-offs incurred by irrigating pasture during the dry season. Although compensatory growth is an efficient mechanism to reduce feed costs and optimize pasture utilization in Brazilian systems (Lopes et al., 2018), improvements in cattle productivity conferred by pasture quality and yield, with consequent reductions in GHG emissions, might outweigh a relatively minor increase in water use, if due regard was taken for local water availability. Indeed, Ruviaro et al. (2015) demonstrated that improving beef cattle pastures from a natural grass baseline to those with greater DMI digestibility reduced GHG emissions, improved FCE and had the lowest CH_4_ and N_2_O emissions; with similar results reported by Eri et al. (2020) as a result of Brazilian pastures being renovated after being affected by sudden death disease.

As shown in Table 8, fertilizers, pesticides and fossil fuel usage followed the same trends as feed and land use—all were reduced by implant use, the magnitude of the reduction increasing with the intensity of implant effects on production. Given the currently low proportion of feedlot cattle, Brazilian beef production is characterized by a relatively low use of nonrenewable mined resource inputs, therefore changes in the total quantities are relatively less than would be expected by implant use in more intensive systems. However, the resource use reductions conferred by implant use are not insignificant, particularly if scaled up to represent regional or national production—a valid extrapolation given the contribution of MG and MGS to national beef production (Cerri et al., 2016).

In 2015, the Brazilian national government committed to reducing GHG emissions by 38% by 2025 (compared to a 2005 baseline), focusing upon the promotion of holistic approaches to land management, sustainable practices and implementation of the Brazilian Forest Code (Federative Republic of Brazil, 2015, 2020). National GHG emissions have declined significantly since 2005, with agriculture’s contribution increasing slightly over time, at 33.6% of the total in 2016, yet total emissions appear to have remained constant since 2009 (Federative Republic of Brazil, 2020). Practices and technologies that allow further reductions in GHG emissions from agriculture are therefore essential to fulfill the commitment made in 2015, with beef production playing an intrinsic role.

The GHG emissions per kg HCW beef within the current study are comparable to those published by Cederberg et al. (2009), Desjardins et al. (2012), Dick et al. (2015), Ruviaro et al. (2015), Pashaei Kamali et al. (2016), Florindo et al. (2017), and de Figueiredo et al. (2017) for Brazilian beef production. Moreover, the relatively limited number of studies that have quantified the effects of implant use within beef production, either alone or in conjunction with other PET, have reported similar results, with reductions in GHG emissions per unit of beef conferred by PET use ranging from 5.8% to 40.3% (Cooprider et al., 2011; Basarab et al., 2012; Capper, 2012; Capper and Hayes, 2012; Stackhouse-Lawson et al., 2012, 2013; Webb, 2018). The positive impacts of using hormone implants, with an 9.4 percent reduction in GHG emissions conferred by implants at the lowest performance enhancing level (LI), rising to 15.8% at the highest level (HI), are shown in Table 8. These are considerable reductions, especially given that they only impact the growing and finishing cattle within the population, having no productivity enhancing effect on cows, heifers and bulls within the supporting herd. To put the potential GHG emissions avoided by implant use into context: using impacts at the medium (MI) level of performance enhancement compared to the NI scenario, scaled up from 1.0 × 10^6^ kg HCW to total Brazilian beef production, would be equivalent to eliminating the exhaust emissions of 62.0 million Brazilian cars, or planting 11.3 billion trees—a considerable environmental bonus.

Mazzetto et al. (2015) assessed the impact of intensifying beef systems, including improving pasture, animal performance and genetic gain, showing that GHG emissions per kg CW could be improved from 2% to 57%, depending on management change. As shown in Tables 1 and 2, the productivity metrics relating to the Brazilian beef operations within the current study reveal various additional opportunities for improvement that would be expected to reduce resource use and GHG emissions. The cow-calf component of beef systems often contributes the greatest proportion of GHG emissions per kg HCW beef, because multiple animals (one or more cows, plus various proportions of mature bulls, replacement heifers and replacement bulls) have to be present in the supporting herd to produce one finished animal. Brazilian beef operations within the current study only weaned ~0.7 calves per breeding cow (Table 1). This represents a significant productivity loss that cannot be compensated further downstream. Considerable environmental gains may therefore be achieved by increasing the number of calves weaned, in combination with improving growth and fertility such that the current age at first calving and calving interval are reduced (Day and Nogueira, 2013; Lobato et al., 2014).

**Figure 2.**
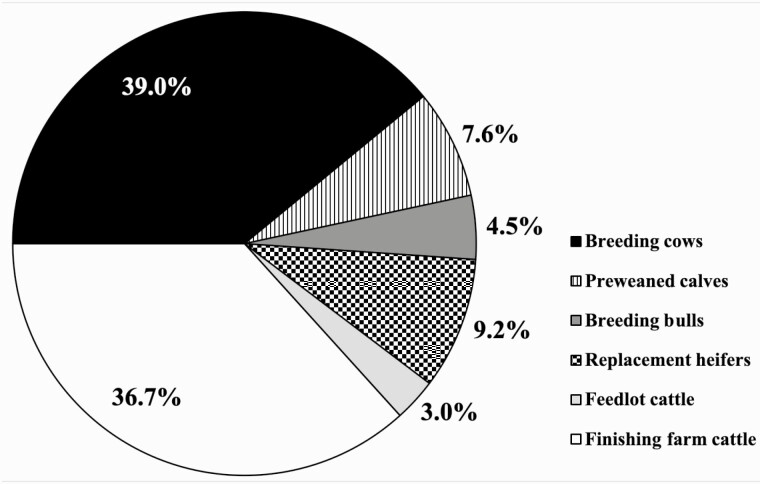
Proportional contributions of cattle groups to total emissions (CO_2_e) per kg of hot carcass weight beef (based on “no implant” population).

In contrast to the results published by Nieto et al. (2018) and Beauchemin et al. (2010) and as shown in Figure 2, the extended age at slaughter of Brazilian beef cattle, confers the burden of a greater proportion of total GHG emissions to growing and finishing cattle, at a total of 39.7% (36.7% for finishing farm cattle plus 3.0% for feedlot cattle). In consequence, considerable opportunities still exist to reduce resource use and greenhouse gas emissions by improving the performance of growing and finishing cattle. As reported by Vasconcelos et al. (2018), improving pasture quality provides another avenue for GHG mitigation. This strategy was highlighted by Ruviaro et al. (2015), who showed that improving pasture productivity could considerably reduce GHG emissions from Brazilian beef production by increasing ADG, with the most productive scenario (cultivated ryegrass plus sorghum supplementation) resulting in a carbon footprint equal to 18.1 kg of CO_2_e per kg of LW gain. Florindo et al. (2017) also evaluated the effects of supplemental feed provided to growing and finishing Brazilian beef cattle and reported that the cattle with the greatest ADG and LW gain per ha, finishing at 510 kg at 20 mo of age, had the lowest GHG emissions (17.09 kg CO_2_e per kg of LW, 45% lower than the control) within the groups studied. Furthermore, in a survey of 40 cattle ranchers within the Brazilian Amazon biome, Bogaerts et al. (2017) reported that farms that participated in sustainability programs with intensified production (greater stocking rates and lesser age at slaughter) had GHG emissions 8.3 kg CO_2_e per kg of CW less than those that did not participate. This 19% decrease increased to 35.8% on farms that had participated in the programs for two years or more.

**Figure 3.**
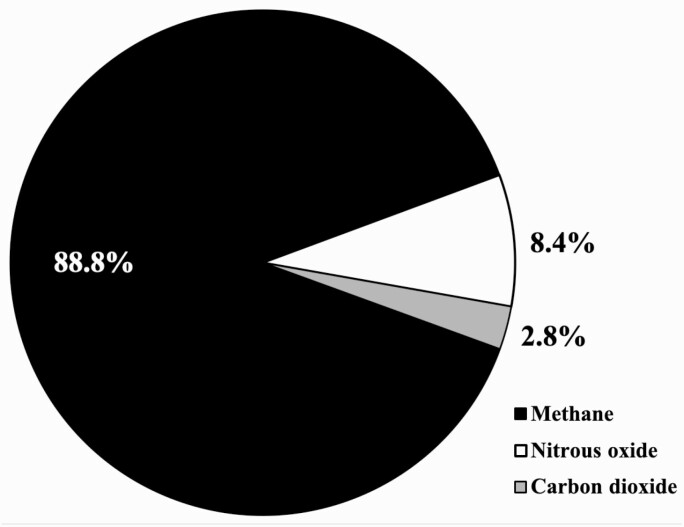
Proportional contributions of different greenhouse gases to total emissions (CO_2_e) per kg of hot carcass weight beef (based on the “no implant” population).

Of the three primary GHG emitted from beef production within the current study, CH_4_ accounted for the greatest share of emissions at 88.8% (Figure 3) with N_2_O at 8.4% and CO_2_ at 2.8%. These proportions are similar to those reported by Bogaerts et al. (2017). Cerri et al. (2016) noted similar results when quantifying the GHG emissions from 22 beef farms in the MG region of Brazil, yet their cited emissions per kg CW were considerably lower, at 9.0–15.5 kg CO_2_e per kg CW. This discrepancy may have been due to the variation in beef herd populations surveyed by Cerri et al. (2016), as the relative proportions of cows and young cattle varied from 0:6,087 to 500:61. The preponderance of CH_4_ within the GHG emissions per kg HCW beef is of importance however, as if the new GWP* metric proposed by Cain et al. (2019), which accounts for atmospheric CH_4_ degradation over time was employed, the magnitude of the difference in GHG emissions between traditional Brazilian beef systems and intensive systems might be significantly reduced (Picasso et al., 2014; Allen et al., 2018; Cain et al., 2019; Lynch, 2019; Lynch et al., 2020). The relatively high GHG emissions per kg of HCW beef (as measured by GWP100) shown in Table 8 should therefore not necessarily be taken as evidence that Brazilian beef production systems have a greater negative environmental impact than other regional systems. Furthermore, as discussed by Bragaglio et al. (2020), the benefits of providing accessory services such as carbon sequestration, enhanced biodiversity and use of human-inedible feeds within primarily pasture-based systems should also be considered.

Carbon sequestration was not included within the current model, primarily due to lack of accurate data on the potential for sequestration in Brazilian soils under varying management strategies. Given the need to report and reduce national GHG emissions data, measuring and benchmarking soil organic carbon is likely to be mandatory for beef production systems across the globe in future, therefore this knowledge gap will need to be addressed and incorporated into modeling scenarios and carbon tools (Bogaerts et al., 2017). Several studies have shown that improving tropical grass productivity results in increased soil carbon stocks, with Maia et al. (2009) citing net atmospheric CO_2_ removals of almost 1 MgC/ha in improved pastures. In an assessment of different Brazilian beef production systems, de Figueiredo et al. (2017) reported that including C sequestration in managed pasture and integrated crop-livestock-forest systems reduced GHG emissions from 9.4 kg to 7.6 kg CO_2_e/kg LW (managed pasture) and 12.6 kg to –28.1 kg CO_2_e/kg LW in the integrated crop-livestock-forest system. It is possible, therefore, that given appropriate pasture management, enhanced sequestration would have an additive effect on reducing GHG emissions.

Any change in management practices or technology use designed to improve system sustainability must have a neutral or positive effect upon economic viability over time. Using data from an earlier review of 37 implant trials (Duckett et al., 1996), Duckett and Pratt (2014) calculated increases in returns of $77/head (at 2014 prices) resulting from implant use, and Duckett and Andrae (2001) reported $93 increases in value per animal in cattle implanted in cow-calf, stocker and feedlot phases. Improving productivity such that resource use and GHG emissions are reduced would be expected to reduce economic costs of production, yet, as reported by Ruviaro et al. (2016) and Pashaei Kamali et al. (2016), management interventions that have the greatest impact on environmental metrics, are not necessarily the most economically beneficial. Using implants in Brazilian beef cattle had positive effects upon the economic viability of beef production systems, as measured by costs and returns per kg of beef (Tables 9 and 10) and per ha (Supplementary Appendix 1). The economic results show that, in addition to a higher return on invested capital, implant use provided gains in terms of economies of scale. As production increased, cash costs were reduced, resulting in a greater margin for the producers within each system (cow-calf through to finishing). The 6.13% increase in kg of HCW beef produced generates a cost reduction of 3.76% and an increase in the return on invested capital of 4.14% on average (Table 10).

**Table 9.** Economic costs of Brazilian beef production per kg HCW beef conferred by implant use at low, medium or high levels of performance enhancement compared to a control (no implants) scenario

	No implants (NI)		Low (LI)		Medium (MI)		High (HI)	
	Cow-calf	Finishing	Cow-calf	Finishing	Cow-calf	Finishing	Cow-calf	Finishing
**Mato Grosso**								
Administration	0.05	0.02	0.04	0.02	0.04	0.02	0.04	0.02
Purchase of animals	0.05	1.09	0.04	0.97	0.04	0.93	0.03	0.89
System maintenance	0.02	0.01	0.02	0.01	0.02	0.01	0.02	0.01
Feed	0.15	0.11	0.14	0.10	0.13	0.10	0.13	0.09
Fuel	0.04	0.02	0.04	0.01	0.03	0.01	0.03	0.01
Labor	0.14	0.03	0.13	0.03	0.12	0.03	0.12	0.03
Veterinary medicines	0.02	0.01	0.01	0.01	0.01	0.01	0.01	0.01
Implants^*a*^	-	-	0.01	0.02	0.01	0.02	0.01	0.01
**Mato Grosso do Sul**								
Administration	0.05	0.10	0.04	0.09	0.04	0.09	0.04	0.09
Purchase of animals	0.04	1.32	0.03	1.26	0.04	1.23	0.03	1.21
System maintenance	0.04	0.02	0.04	0.02	0.04	0.02	0.03	0.02
Feed	0.08	0.14	0.07	0.14	0.07	0.14	0.07	0.13
Fuel	0.06	0.04	0.05	0.04	0.05	0.04	0.05	0.03
Labor	0.11	0.16	0.10	0.15	0.10	0.15	0.09	0.15
Veterinary medicines	0.02	0.01	0.02	0.01	0.02	0.01	0.02	0.01
Implants^*a*^	-	-	0.01	0.02	0.01	0.02	0.01	0.02
**Goias**	**Feedlot**		**Feedlot**		**Feedlot**		**Feedlot**	
Administration	0.04		0.03		0.03		0.03	
Purchase of animals	3.41		3.03		2.89		2.76	
System maintenance	0.02		0.02		0.01		0.01	
Feed	1.30		1.10		1.02		0.95	
Fuel	0.02		0.02		0.02		0.02	
Labor	0.09		0.08		0.07		0.07	
Veterinary medicines	0.00		0.00		0.00		0.00	
Implants^*a*^	-		0.01		0.01		0.01	

^*a*^See Table 6 for implant costs per head.

**Table 10.** Economic effects of implant use at low, medium and high levels of performance enhancement compared to no implants in Brazilian beef systems—revenue, costs, margin, return on investment and productivity per kg of HCW beef

	No implants (NI)		Low (LI)		Medium (MI)		High (HI)	
	Cow-calf	Finishing	Cow-calf	Finishing	Cow-calf	Finishing	Cow-calf	Finishing
**Mato Grosso**								
Revenue^*a*^	1.61	1.98	1.50	2.04	1.46	2.06	1.45	2.08
Cash cost^*a*^	0.46	1.29	0.43	1.17	0.41	1.12	0.40	1.06
Cash cost + depreciation^*a*^	0.71	1.39	0.65	1.25	0.63	1.20	0.61	1.14
Gross margin^*a*^	1.15	0.70	1.07	0.87	1.05	0.95	1.05	1.02
Net margin^*a*^	0.90	0.60	0.85	0.79	0.83	0.87	0.84	0.95
Return on real investment^*b*^	3.48	1.54	3.51	1.75	3.54	1.85	3.61	1.96
Return on real investment^*c*^	2.26	1.43	2.30	1.63	2.33	1.72	2.37	1.83
**Mato Grosso do Sul**								
Revenue^*a*^	1.41	2.12	1.28	2.23	1.29	2.28	1.20	2.31
Cash cost^*a*^	0.40	1.80	0.36	1.73	0.36	1.70	0.33	1.66
Cash cost + depreciation^*a*^	0.72	2.19	0.65	2.10	0.65	2.06	0.60	2.02
Gross margin^*a*^	1.02	0.32	0.92	0.49	0.93	0.58	0.86	0.65
Net margin^*a*^	0.69	-0.07	0.63	0.13	0.64	0.22	0.60	0.30
Return on real investment^*b*^	3.56	1.18	3.55	1.28	3.58	1.34	3.60	1.39
Return on real investment^*c*^	1.97	0.97	1.98	1.06	1.99	1.11	2.00	1.15
**Goias**	**Feedlot**		**Feedlot**		**Feedlot**		**Feedlot**	
Revenue^*a*^	4.85		4.99		5.05		5.11	
Cash cost^*a*^	4.87		4.27		4.05		3.85	
Cash cost + depreciation^*a*^	4.98		4.37		4.14		3.94	
Gross margin^*a*^	-0.02		0.72		1.00		1.26	
Net margin^*a*^	-0.13		0.62		0.91		1.17	
Return on real investment^*b*^	1.00		1.17		1.25		1.33	
Return on real investment^*c*^	0.97		1.14		1.22		1.30	

^*a*^US$ per kg HCW beef.

^*b*^Revenue/cash cost.

^*c*^Revenue/(cash cost + depreciation).

Economic viability ultimately depends on a continuing demand and willingness to pay for the product and therefore meat quality plays a key role. Meat presentation, color and price were cited by Barcellos et al. (2019) as being the three most important attributes for Brazilian beef consumers, with little attention paid to leanness or marbling. This may be somewhat advantageous, as researchers investigating implant use in Bos indicus cattle, which tend to have lean carcasses, reported reduced meat tenderness from implanted cattle compared to controls (Thompson et al., 2008a, 2008b). The effects of implant use on meat tenderness are somewhat inconclusive according to Duckett and Pratt (2014), yet Platter et al. (2003) showed that consumers preferred steaks from nonimplanted cattle, although using implants early in life (branding, weaning and backgrounding) did not affect consumer satisfaction. However, given that Delgado et al. (2006) concluded that Brazilian consumers were able to differentiate between tender and tough steaks, any practice or technology that increases toughness might have negative economic consequences, therefore mitigation measures should be implemented.

The improvements in environmental and economic sustainability conferred by implant use within the current study reflect results of previous analyses (Basarab et al., 2012; Capper, 2012, 2013; Capper and Hayes, 2012; Stackhouse-Lawson et al., 2012; White and Capper, 2014; Webb et al., 2017), and strongly support the role of these technologies in reducing resource use and improving economic returns per kg of HCW beef. However, sustainability is triumvirate in nature, therefore the third component—social acceptability—must be in place. Nebulous yet negative consumer concerns regarding PET use are more difficult to address. As discussed by Smith and Johnson (2020), the safety of hormonal implants, both for the implanted cattle and the end consumers of meat from the animals, is ensured by thorough testing, setting maximum residue levels and monitoring residues in tissues. In the United States, for example, no residue violations have occurred in over 11 years. Furthermore, the increases in individual hormone concentrations in meat from implanted animals are negligible compared to endogenous synthesis within the human body (Palacios et al., 2020) and only 0.1–10% of the quantity ingested is absorbed into the bloodstream (Doyle, 2000). There appears to be no scientific justification for consumer concerns, yet the marketplace popularity of ASF labeled as being raised without hormones led Aboagye et al. (2021) to question whether PET use might reduce consumers’ willingness to buy implanted beef, although it was noted that claims about purchasing (e.g., only buying natural or organic products) were often confounded by price and so not reflected by purchasing behaviors. Concerns over the environmental impacts of ASF are often cited as a rationale for reducing or eliminating their consumption—the question of whether reductions in resource use and GHG emissions conferred by implant use would outweigh potential (albeit unfounded) consumer concerns regarding safety or human health perceptions, has yet to be answered within the literature.

The potential impacts of PET use on global trade should also be considered, given Brazil’s role as a significant beef exporter. After β-adrenergic agonists (βAA) were approved for use in Brazil, and implants approved in Argentina, some countries responded by demanding beef from cattle that were not given these technologies, thereby requiring supply chain segregation (Millen and Arrigoni, 2013). Given this segregation precedent, implant use might not be a significant issue if it were deemed acceptable in cattle destined for the domestic beef market, yet potential economic impacts of export market accessibility, along with the practical feasibility of supply chain segregation should be considered. Europe is Brazil’s third-largest export market—in 2019, Brazilian beef exports to Europe were valued at $619 million USD (Statistica, 2021), equal to almost 100,000 tons of CW. If this market were lost, it would have significant negative effects on domestic oversupply and therefore, economic viability at the producer level. However, a niche market based on PET-free beef might offer opportunities for some producers within the Brazilian system, as discussed by Aboagye et al. (2021).

The use of implants in Brazilian beef cattle provides clear and significant opportunities to improve resource use, GHG emissions and the economic viability of beef production systems, which all contribute to improving overall system sustainability. Given Brazil’s significant contribution to global beef production, with consequent implications for its role in anthropogenic climate change, it is crucial for beef producers to demonstrate dedication to improving environmental impacts. This may be achieved, in part, by simply improving production efficiency, although this must be done in a conscious manner to make the best use of resources, including previously deforested land. Changes in management practice and PET adoption must be executed in a manner that do not lead to negative economic consequences, e.g., limited access to export markets, or impaired consumer confidence. Brazilian beef producers must therefore focus on improving all three facets of sustainability (environmental responsibility, economic viability and social acceptability) in a holistic manner.

## Supplementary Material

txab144_suppl_Supplementary_Materials

## References

[CIT0001] Aboagye, I. A., M. R. C. Cordeiro, T. A. McAllister, and K. H. Ominski. 2021. Productivity-enhancing technologies. Can consumer choices affect the environmental footprint of beef? Sustainability 13(8). doi:10.3390/su13084283

[CIT0002] Al-Husseini, W., C. Gondro, K. Quinn, L. M. Cafe, R. M. Herd, J. P. Gibson, P. L. Greenwood, and Y. Chen. 2014. Hormonal growth implants affect feed efficiency and expression of residual feed intake-associated genes in beef cattle. Anim. Prod. Sci. 54(5):550–556.

[CIT0003] Alford, A. R., R. S. Hegarty, P. F. Parnell, O. J. Cacho, R. M. Herd, and G. R. Griffith. 2006. The impact of breeding to reduce residual feed intake on enteric methane emissions from the Australian beef industry. Aust. J. Exp. Agric. 46(7):813–820.

[CIT0004] Allen, M. R., K. P. Shine, J. S. Fuglestvedt, R. J. Millar, M. Cain, D. J. Frame, and A. H. Macey. 2018. A solution to the misrepresentations of CO_2_-equivalent emissions of short-lived climate pollutants under ambitious mitigation. NPJ Clim. Atmos. Sci. 1(1):16. doi:10.1038/s41612-018-0026-8

[CIT0005] AMTS . 2018. Cattle Pro. Ithaca, NY: Cornell Research Foundation.

[CIT0006] Aroeira, C. N., V. Feddern, V. Gressler, C. J. Contreras-Castillo, and D. L. Hopkins. 2021. A review on growth promoters still allowed in cattle and pig production. Livest. Sci. 247:104464. doi:10.1016/j.livsci.2021.104464

[CIT0007] Barcellos, V. C., C. Mottin, R. M. d. Prado, T. Schenkel, C. Viana, A. C. P. Vital, L. d. S. Bersot, C. Sañudo, and I. N. d. Prado. 2019. How the perception of quality for beef evaluated by the buyer at the time of purchase: study in three Brazilian cities of different sizes - Curitiba, Campo Mourão and Palotina. Acta Sci. 41.

[CIT0008] Basarab, J., V. Baron, Ó. López-Campos, J. Aalhus, K. Haugen-Kozyra, and E. Okine. 2012. Greenhouse gas emissions from calf- and yearling-fed beef production systems, with and without the use of growth promotants. Animals. 2:195–220. doi:10.3390/ani2020195 26486917 PMC4494322

[CIT0009] Beauchemin, K. A., H. Janzen, S. M. Little, T. A. McAllister, and S. M. McGinn. 2010. Life cycle assessment of greenhouse gas emissions from beef production in western Canada: a case study. Agric. Syst. 103:371–379. 10.3168/jds.2011-5229 22916922

[CIT0010] Beauchemin, K. A., H. H. Janzen, S. M. Little, T. A. McAllister, and S. M. McGinn. 2011. Mitigation of greenhouse gas emissions from beef production in western Canada – evaluation using farm-based life cycle assessment. Anim. Feed Sci. Tech. 166–167:663–677.

[CIT0011] Beck, P., B. Barham, J. Apple, W. Whitworth, M. Miller, and S. Gadberry. 2012. Effect of age entering feedlot and implant regimen on finishing system profitability. Prof. Anim. Sci. 28(1):32–40. doi:10.15232/S1080-7446(15)30313-2

[CIT0012] Beck, P., T. Hess, D. Hubbell, G. D. Hufstedler, B. Fieser, and J. Caldwell. 2014. Additive effects of growth promoting technologies on performance of grazing steers and economics of the wheat pasture enterprise. J. Anim. Sci. 92:1219–1227. doi:10.2527/jas.2013-7203 24492552

[CIT0013] Bogaerts, M., L. Cirhigiri, I. Robinson, M. Rodkin, R. Hajjar, C. Costa Junior, and P. Newton. 2017. Climate change mitigation through intensified pasture management: estimating greenhouse gas emissions on cattle farms in the Brazilian Amazon. J. Clean. Prod. 162:1539–1550. doi:10.1016/j.jclepro.2017.06.130

[CIT0014] Bragaglio, A., A. Braghieri, C. Pacelli, and F. Napolitano. 2020. Environmental impacts of beef as corrected for the provision of ecosystem services. Sustainability 12(9). doi:10.3390/su12093828

[CIT0015] Cain, M., J. Lynch, M. R. Allen, J. S. Fuglestvedt, D. J. Frame, and A. H. Macey. 2019. Improved calculation of warming-equivalent emissions for short-lived climate pollutants. NPJ Clim. Atmos. Sci. 2:29. doi:10.1038/s41612-019-0086-4 31656858 PMC6814445

[CIT0016] Camargo, G. G. T., M. R. Ryan, and T. L. Richard. 2013. Energy use and greenhouse gas emissions from crop production using the farm energy analysis tool. Bioscience. 63:263–273.

[CIT0017] Cappellozza, B. I., A. C. Velasco, C. Tongu, G. Moraes, R. Dib, and R. Cervieri. 2020. Effects of supplement amount, with or without calcium salts of fatty acids, on growth performance and intake behavior of grazing *Bos indicus* bulls. Transl. Anim. Sci. 4:799–808. doi:10.1093/tas/txz190 33554051 PMC6999045

[CIT0018] Capper, J. L . 2011. The environmental impact of beef production in the United States: 1977 compared with 2007. J. Anim. Sci. 89:4249–4261. doi:10.2527/jas.2010-3784 21803973

[CIT0019] Capper, J. L . 2012. Is the grass always greener? Comparing resource use and carbon footprints of conventional, natural and grass-fed beef production systems. Animals. 2:127–143. doi:10.3390/ani2020127 26486913 PMC4494320

[CIT0020] Capper, J. L . 2013. The environmental and economic impact of steroid implant and beta-adrenergic agonist use within U.S. beef production. Proceedings of the ADSA-ASAS Joint Annual Meeting, Indianapolis, IN, USA.

[CIT0021] Capper, J. L . 2020. Opportunities and challenges in animal protein industry sustainability: the battle between science and consumer perception. Anim. Front. 10:7–13. doi:10.1093/af/vfaa034 33150006 PMC7596800

[CIT0022] Capper, J. L., and R. A. Cady. 2020. The effects of improved performance in the U.S. dairy cattle industry on environmental impacts between 2007 and 2017. J. Anim. Sci. 98(1). doi:10.1093/jas/skz291 PMC6978902 31622980

[CIT0023] Capper, J. L., and D. J. Hayes. 2012. The environmental and economic impact of removing growth-enhancing technologies from United States beef production. J. Anim. Sci. 90:3527–3537. doi:10.2527/jas.2011-4870 22665660

[CIT0024] Cardoso, A. S., A. Berndt, A. Leytem, B. J. R. Alves, I. d. N. O. de Carvalho, L. H. de Barros Soares, S. Urquiaga, and R. M. Boddey. 2016. Impact of the intensification of beef production in Brazil on greenhouse gas emissions and land use. Agric. Syst. 143:86–96. doi:10.1016/j.agsy.2015.12.007

[CIT0025] Casey, N. H., and P. J. Holden. 2006. Quantification of GHG emissions from sucker-beef production in Ireland. Agric. Syst. 90:79–98.

[CIT0026] Cederberg, C., D. Meyer, and A. Flysjo. 2009. Life cycle inventory of greenhouse gas emissions and use of land and energy in Brazilian beef production. Gothenburg, Sweden: The Swedish Institute for Food and Biotechnology.

[CIT0027] Cederberg, C., U. M. Persson, K. Neovius, S. Molander, and R. Clift. 2011. Including carbon emissions from deforestation in the carbon footprint of Brazilian beef. Environ. Sci. Technol. 45:1773–1779. doi:10.1021/es103240z 21280649

[CIT0028] Cerri, C. E. P., C. C. Cerri, S. M. F. Maia, M. R. Cherubin, B. J. Feigl, and R. Lal. 2018. Reducing Amazon deforestation through agricultural intensification in the Cerrado for advancing food security and mitigating climate change. Sustainability 10(4). doi:10.3390/su10040989

[CIT0029] Cerri, C. C., C. S. Moreira, P. A. Alves, G. S. Raucci, B. de Almeida Castigioni, F. F. C. Mello, D. G. P. Cerri, and C. E. P. Cerri. 2016. Assessing the carbon footprint of beef cattle in Brazil: a case study with 22 farms in the State of Mato Grosso. J. Clean. Prod. 112:2593–2600. doi:10.1016/j.jclepro.2015.10.072

[CIT0030] Cleale, R. M., D. R. Hilbig, T. H. Short, S. H. Sweiger, and T. Gallery. 2018. Effects of Synovex One Grass, Revalor-G, or Encore implants on performance of steers grazing for up to 200 days. Prof. Anim. Sci. 34(2):192–201. doi:10.15232/pas.2017-01685

[CIT0031] Cohn, A. S., A. Mosnier, P. Havlík, H. Valin, M. Herrero, E. Schmid, M. O’Hare, and M. Obersteiner. 2014. Cattle ranching intensification in Brazil can reduce global greenhouse gas emissions by sparing land from deforestation. Proc. Natl. Acad. Sci. U. S. A. 111:7236–7241. doi:10.1073/pnas.1307163111 24778243 PMC4034253

[CIT0032] Coltro, L., A. L. Mourad, R. M. Kletecke, T. A. Mendonça, and S. P. M. Germer. 2009. Assessing the environmental profile of orange production in Brazil. Int. J. Life Cycle Assess. 14(7):656–664. doi:10.1007/s11367-009-0097-1

[CIT0033] Cooprider, K. L., F. M. Mitloehner, T. R. Famula, E. Kebreab, Y. Zhao, and V. Eenennaam. 2011. Feedlot efficiency implications on GHG emissions and sustainability. J. Anim. Sci. 89:2643–2656. 21398565 10.2527/jas.2010-3539

[CIT0034] Cotton Inc . 2012. Life cycle assessment of cotton fiber and fabric. Accessed June 1, 2021. https://cottonleads.org/wp-content/uploads/2018/02/Cotton-LEADS-LCA-2012.pdf

[CIT0035] Day, M. L., and G. P. Nogueira. 2013. Management of age at puberty in beef heifers to optimize efficiency of beef production. Anim. Front. 3(4):6–11. doi:10.2527/af.2013-0027

[CIT0036] de Figueiredo, E. B., S. Jayasundara, R. de Oliveira Bordonal, T. T. Berchielli, R. A. Reis, C. Wagner-Riddle, and N. La Scala Jr. 2017. Greenhouse gas balance and carbon footprint of beef cattle in three contrasting pasture-management systems in Brazil. J. Clean. Prod. 142:420–431. doi:10.1016/j.jclepro.2016.03.132

[CIT0037] de Oliveira Silva, R., L. G. Barioni, T. Z. Albertini, V. Eory, C. F. E. Topp, F. A. Fernandes, and D. Moran. 2015. Developing a nationally appropriate mitigation measure from the greenhouse gas GHG abatement potential from livestock production in the Brazilian Cerrado. Agric. Syst. 140:48–55. doi:10.1016/j.agsy.2015.08.011

[CIT0038] de Oliveira Silva, R., L. G. Barioni, J. A. J. Hall, M. Folegatti Matsuura, T. Zanett Albertini, F. A. Fernandes, and D. Moran. 2016. Increasing beef production could lower greenhouse gas emissions in Brazil if decoupled from deforestation. Nat. Clim. Chang. 6(5):493–497. doi:10.1038/nclimate2916

[CIT0039] de Vries, M., C. E. van Middelaar, and I. J. M. de Boer. 2015. Comparing environmental impacts of beef production systems: a review of life cycle assessments. Livest. Sci. 178:279–288. doi:10.1016/j.livsci.2015.06.020

[CIT0040] Delgado, E. F., A. P. Aguiar, E. M. M. Ortega, M. H. F. Spoto, and C. J. C. Castillo. 2006. Brazilian consumers’ perception of tenderness of beef steaks classified by shear force and taste. Sci. Agric. 63:232–239.

[CIT0041] Desjardins, R. L., D. E. Worth, X. P. C. Vergé, D. Maxime, J. Dyer, and D. Cerkowniak. 2012. Carbon footprint of beef cattle. Sustainability 4(12). doi:10.3390/su4123279

[CIT0042] Dick, M., M. Abreu da Silva, and H. Dewes. 2015. Life cycle assessment of beef cattle production in two typical grassland systems of southern Brazil. J. Clean. Prod. 96:426–434. doi:10.1016/j.jclepro.2014.01.080

[CIT0043] Domke, G. M., S. N. Oswalt, B. F. Walters, and R. S. Morin. 2020. Tree planting has the potential to increase carbon sequestration capacity of forests in the United States. Proc. Natl. Acad. Sci. U. S. A. 117:24649–24651. doi:10.1073/pnas.2010840117 32958649 PMC7547226

[CIT0044] Doreau, M., M. S. Corson, and S. G. Wiedemann. 2012. Water use by livestock: a global perspective for a regional issue? Anim. Front. 2:9–16.

[CIT0045] Doyle, E . 2000. Human safety of hormone implants used to promote growth in cattle a review of the scientific literature. Briefings: Food Research Institute. Madison WI, USA: University of Wisconsin.

[CIT0046] Duckett, S. K., and J. G. Andrae. 2001. Implant strategies in an integrated beef production system. J. Anim. Sci. 79(suppl_E):E110–E117. doi:10.2527/jas2001.79E-SupplE110x

[CIT0047] Duckett, S., and S. L. Pratt. 2014. Meat science and muscle biology symposium – anabolic implants and meat quality. J. Anim. Sci. 92:3–9. 24243897 10.2527/jas.2013-7088

[CIT0048] Duckett, S. K., D. G. Wagner, F. N. Owens, H. G. Dolezal, and D. R. Gill. 1996. Effects of estrogenic and androgenic implants on performance, carcass traits, and meat tenderness in feedlot steers: a review. Prof. Anim. Sci. 12:205–214.

[CIT0049] Eisler, M. C., M. R. Lee, J. F. Tarlton, G. B. Martin, J. Beddington, J. A. Dungait, H. Greathead, J. Liu, S. Mathew, H. Miller, et al. 2014. Agriculture: steps to sustainable livestock. Nature 507:32–34. doi:10.1038/507032a 24605375

[CIT0050] Eri, M., C. A. d. Silva Junior, M. Lima, N. La Scala Júnior, J. F. d. Oliveira-Júnior, P. E. Teodoro, G. F. Capristo-Silva, G. Caione, and C. A. Peres. 2020. Capitalizing on opportunities provided by pasture sudden death to enhance livestock sustainable management in Brazilian Amazonia. Environ. Dev. 33:100499. doi:10.1016/j.envdev.2020.100499

[CIT0051] FAO . 2006. Fertilizer Use by Crop in Brazil. Rome, Italy: FAO, Online: Land and Plant Nutrition Management Service - Land and Water Development Division.

[CIT0052] FAO . 2013. Tackling climate change through livestock – a global assessment of emissions and mitigation opportunities. Rome, Italy: Food and Agriculture Organization of the United Nations.

[CIT0053] FAO . 2021. FAOSTAT. Accessed February 17, 2021.http://faostat.fao.org

[CIT0054] Fearnside, P. M . 2005. Deforestation in Brazilian Amazonia: history, rates, and consequences. Conserv Biol. 19(3):680–688. doi:10.1111/j.1523-1739.2005.00697.x

[CIT0055] Federative Republic of Brazil . 2015. Intended nationally determined contribution towards achieving the objective of the United Nations Framework Convention on Climate Change. Accessed April 23, 2021. http://www4.unfccc.int/submissions/INDC/Published%20Documents/Brazil/1/BRAZIL%20iNDC%20english%20FINAL.pdf

[CIT0056] Federative Republic of Brazil . 2020. Fourth biennial update report of Brazil to the United Nations Framework Convention on Climate Change. Brazil: Ministry of Foreign Affairs and Ministry of Science, Technology and Innovations.

[CIT0057] Feedipedia . 2021. Citrus pulp, fresh. Accessed March 1, 2021. https://www.feedipedia.org/node/679

[CIT0058] Ferraz, J. B. S., and P. E. d. Felício. 2010. Production systems – an example from Brazil. Meat Sci. 84(2):238–243. doi:10.1016/j.meatsci.2009.06.006 20374781

[CIT0059] Flach, R., R. Skalský, C. Folberth, J. Balkovič, K. Jantke, and U. A. Schneider. 2020. Water productivity and footprint of major Brazilian rainfed crops – a spatially explicit analysis of crop management scenarios. Agric. Water Manage. 233:105996. doi:10.1016/j.agwat.2019.105996

[CIT0060] Fliehr, O . 2013. Analysis of transportation and logistics processes for soybeans in Brazil. A case study of selected production regions. Thünen Working Paper 4. Braunschweig, Germany: Thünen Institute of Farm Economics.

[CIT0061] Florindo, T. J., G. I. B. de Medeiros Florindo, E. Talamini, J. S. da Costa, and C. F. Ruviaro. 2017. Carbon footprint and life cycle costing of beef cattle in the Brazilian Midwest. J. Clean. Prod. 147:119–129. doi:10.1016/j.jclepro.2017.01.021

[CIT0062] Franco Junior, N. C., M. Milan, and T. L. Romanelli. 2014. Energy demand in citrus production under varied operational efficiency values. Eng. Agríc. 34:746–754.

[CIT0063] Glensor, K., and M. R. Muñoz. 2019. Life-cycle assessment of Brazilian transport biofuel and electrification pathways. Sustainability 11(22). doi:10.3390/su11226332

[CIT0064] Hyland, J. J., D. Styles, D. L. Jones, and A. P. Williams. 2016. Improving livestock production efficiencies presents a major opportunity to reduce sectoral greenhouse gas emissions. Agric. Syst. 147:123–131. doi:10.1016/j.agsy.2016.06.006

[CIT0065] Instituto Brasileiro de Geografia e Estatistica . 2021. Census of agriculture. Accessed February 2, 2021. https://www.ibge.gov.br/en/statistics/economic/agriculture-forestry-and-fishing/21929-2017-2017-censo-agropecuario-en.html?edicao=25839&t=resultados

[CIT0066] Instituto Brasileiro de Geografia e Estatística . 2021. Municipal livestock research. Accessed January 20, 2021. https://sidra.ibge.gov.br/tabela/3939

[CIT0067] IPCC . 2006. IPCC Guidelines for National Greenhouse Gas Inventories. Kanagawa, Japan: Institute for Global Environmental Strategies (IGES) for the IPCC.

[CIT0068] IPCC . 2013. Climate Change 2013 – the physical science basis. Contribution of Working Group I to the Fourth Assessment Report of the Intergovernmental Panel on Climate Change, Geneva, Switzerland: IPCC Secretariat.

[CIT0069] IPCC . 2019. 2019 refinement to the 2006 IPCC Guidelines for National Greenhouse Gas Inventories. vol. 4. Agriculture, forestry and other land use. Geneva, Switzerland: IPCC.

[CIT0070] Jank, L., S. C. Barrios, C. B. do Valle, R. M. Simeão, and G. F. Alves. 2014. The value of improved pastures to Brazilian beef production. Crop Pasture Sci. 65(11):1132–1137.

[CIT0071] Johnson, B. J., F. R. B. Ribeiro, and J. L. Beckett. 2013. Application of growth technologies in enhancing food security and sustainability. Anim. Front. 3(3):8–13. doi:10.2527/af.2013-0018

[CIT0072] Kaspar, H. F., and J. M. Tiedje. 1981. Dissimilatory reduction of nitrate and nitrite in the bovine rumen: nitrous oxide production and effect of acetylene. Appl. Environ. Microbiol. 41:705–709. doi:10.1128/aem.41.3.705-709.1981 7224631 PMC243764

[CIT0073] Kirchgessner, M., W. Windisch, H. L. Muller, and M. Kreuzer. 1991. Release of methane and of carbon dioxide by dairy cattle. Agribiol. Res. 44:2–9.

[CIT0074] Latawiec, A. E., B. B. Strassburg, J. F. Valentim, F. Ramos, and H. N. Alves-Pinto. 2014. Intensification of cattle ranching production systems: socioeconomic and environmental synergies and risks in Brazil. Animal 8:1255–1263. doi:10.1017/S1751731114001566 26263189

[CIT0075] Lathuillière, M. J., K. Solvik, M. N. Macedo, J. Graesser, E. J. Miranda, E. G. Couto, and M. S. Johnson. 2019. Cattle production in Southern Amazonia: implications for land and water management. Environ. Res. Lett. 14:114025. doi:10.1088/1748-9326/ab30a7

[CIT0076] Legesse, G., K. A. Beauchemin, K. H. Ominski, E. J. McGeough, R. Kroebel, D. MacDonald, S. M. Little, and T. A. McAllister. 2016. Greenhouse gas emissions of Canadian beef production in 1981 as compared with 2011. Anim. Prod. Sci. 56(3):153–168. doi:10.1071/AN15386

[CIT0077] Lesschen, J. P., G. L. Velthof, W. de Vries, and J. Kros. 2011. Differentiation of nitrous oxide emission factors for agricultural soils. Environ. Pollut. 159:3215–3222. doi:10.1016/j.envpol.2011.04.001 21531058

[CIT0078] Lobato, J. F., A. K. Freitas, T. Devincenzi, L. L. Cardoso, J. U. Tarouco, R. M. Vieira, D. R. Dillenburg, and I. Castro. 2014. Brazilian beef produced on pastures: sustainable and healthy. Meat Sci. 98:336–345. doi:10.1016/j.meatsci.2014.06.022 25017318

[CIT0079] Lopes, R. B., M. E. A. Canozzi, L. C. Canellas, F. A. L. Gonzalez, R. F. Corrêa, P. R. R. X. Pereira, and J. O. J. Barcellos. 2018. Bioeconomic simulation of compensatory growth in beef cattle production systems. Livest. Sci. 216:165–173. doi:10.1016/j.livsci.2018.08.011

[CIT0080] Lupo, C. D., D. E. Clay, J. L. Benning, and J. J. Stone. 2013. Life-cycle assessment of the beef cattle production system for the northern great plains, USA. J. Environ. Qual. 42:1386–1394. doi:10.2134/jeq2013.03.0101 24216416

[CIT0081] Lynch, J . 2019. Availability of disaggregated greenhouse gas emissions from beef cattle production: a systematic review. Environ. Impact Assess. Rev. 76:69–78. doi:10.1016/j.eiar.2019.02.003 31388221 PMC6684367

[CIT0082] Lynch, J., M. Cain, R. Pierrehumbert, and M. Allen. 2020. Demonstrating GWP*: a means of reporting warming-equivalent emissions that captures the contrasting impacts of short- and long-lived climate pollutants. Environ. Res. Lett. 15(4):044023. doi:10.1088/1748-9326/ab6d7e 32395177 PMC7212016

[CIT0083] Maçeira, P. M., R. C. Souza, F. L. C. Oliveira, and V. Oliveira. 2017. Electricity consumption forecast for the Brazilian residential sector: a bottom-up approach ISF 2017, Cairns, Australia. Rio de Janeiro, Brazil: Pontifical Catholic University of Rio de Janeiro.

[CIT0084] Maia, S. M. F., S. M. Ogle, C. E. P. Cerri, and C. C. Cerri. 2009. Effect of grassland management on soil carbon sequestration in Rondônia and Mato Grosso states, Brazil. Geoderma 149(1):84–91. doi:10.1016/j.geoderma.2008.11.023

[CIT0085] Marli, M . 2018. Brazilian economy consumes 6 liters of water per each R$1 produced. Accessed March 17, 2021. https://agenciadenoticias.ibge.gov.br/en/agencia-news/2184-news-agency/news/20473-brazilian-economy-consumes-6-liters-of-water-per-each-r-1-produced

[CIT0086] Martha, G. B., E. Alves, and E. Contini. 2012. Land-saving approaches and beef production growth in Brazil. Agric. Syst. 110:173–177.

[CIT0087] Matsunaga, M., P. F. Bemelmans, P. E. N. d. Toledo, R. D. Dulley, H. Okawa, and I. A. Pedroso. 1976. Cost of production methodology utilized by the IEA. Agric. São Paulo 23:123–139.

[CIT0088] Mazzetto, A. M., B. J. Feigl, R. L. M. Schils, C. E. P. Cerri, and C. C. Cerri. 2015. Improved pasture and herd management to reduce greenhouse gas emissions from a Brazilian beef production system. Livest. Sci. 175:101–112. doi:10.1016/j.livsci.2015.02.014

[CIT0089] Millen, D. D., and M. D. B. Arrigoni. 2013. Drivers of change in animal protein production systems: changes from ‘traditional’ to ‘modern’ beef cattle production systems in Brazil. Anim. Front. 3(3):56–60. doi:10.2527/af.2013-0025

[CIT0090] Millen, D. D., R. D. L. Pacheco, P. M. Meyer, P. H. M. Rodrigues, and M. De Beni Arrigoni. 2011. Current outlook and future perspectives of beef production in Brazil. Anim. Front. 1(2):46–52. doi:10.2527/af.2011-0017

[CIT0091] Mogensen, L., T. Kristensen, N. I. Nielsen, P. Spleth, M. Henriksson, C. Swensson, A. Hessle, and M. Vestergaard. 2015. Greenhouse gas emissions from beef production systems in Denmark and Sweden. Livest. Sci. 174:126–143. doi:10.1016/j.livsci.2015.01.021

[CIT0092] Murphy, B., P. Crosson, A. K. Kelly, and R. Prendiville. 2017. An economic and greenhouse gas emissions evaluation of pasture-based dairy calf-to-beef production systems. Agric. Syst. 154:124–132. doi:10.1016/j.agsy.2017.03.007

[CIT0093] Nguyen, T. T., M. Doreau, M. Eugène, M. S. Corson, F. Garcia-Launay, G. Chesneau, and H. M. van der Werf. 2013. Effect of farming practices for greenhouse gas mitigation and subsequent alternative land use on environmental impacts of beef cattle production systems. Animal 7:860–869. doi:10.1017/S1751731112002200. 23190866

[CIT0094] Nieto, M. I., O. Barrantes, L. Privitello, and R. Reiné. 2018. Greenhouse gas emissions from beef grazing systems in semi-arid rangelands of Central Argentina. Sustainability 10:4228–4249. doi:10.3390/su10114228

[CIT0095] North Carolina Soybean Producers Association Inc . 2014. Uses of soybeans. Accessed May 23, 2016. http://ncsoy.org/media-resources/uses-of-soybeans/

[CIT0096] Ogino, A., K. Kaku, T. Osada, and K. Shimada. 2004. Environmental impacts of the Japanese beef-fattening system with different feeding lengths as evaluated by a life-cycle assessment method. J. Anim. Sci. 82:2115–2122. doi:10.2527/2004.8272115x. 15309959

[CIT0097] Oliveira, P. P. A., A. Berndt, A. F. Pedroso, T. C. Alves, J. R. M. Pezzopane, L. S. Sakamoto, F. L. Henrique, and P. H. M. Rodrigues. 2020. Greenhouse gas balance and carbon footprint of pasture-based beef cattle production systems in the tropical region (Atlantic Forest biome). Animal 14(S3):s427–s437. doi:10.1017/S1751731120001822 32829724

[CIT0098] Opio, C., P. Gerber, T. Vellinga, M. MacLeod, A. Falcucci, B. Henderson, A. Mottet, G. Tempio, and H. Steinfeld. 2013. Greenhouse gas emissions from ruminant supply chains: a global life cycle assessment. Rome, Italy: Food and Agriculture Organisation of the United Nations (FAO).

[CIT0099] Paisley, S. I., G. W. Horn, C. J. Ackerman, B. A. Gardner, and D. S. Secrist. 1999. Effects of implants on daily gains of steers wintered on dormant native tallgrass prairie, subsequent performance, and carcass characteristics2. J. Anim. Sci. 77(2):291–299. doi:10.2527/1999.772291x 10100656

[CIT0100] Palacios, O. M., H. N. Cortes, B. H. Jenks, and K. C. Maki. 2020. Naturally occurring hormones in foods and potential health effects. Toxicol. Res. Appl. 4. doi:10.1177/2397847320936281

[CIT0101] Parr, S. L., K. Y. Chung, M. L. Galyean, J. P. Hutcheson, N. DiLorenzo, K. E. Hales, M. L. May, M. J. Quinn, D. R. Smith, and B. J. Johnson. 2010. Performance of finishing beef steers in response to anabolic implant and zilpaterol hydrochloride supplementation. J. Anim. Sci. 89:560–570. doi:10.2527/jas.2010-3101 20935134

[CIT0102] Pashaei Kamali, F., A. van der Linden, M. P. M. Meuwissen, G. C. Malafaia, A. G. J. M. Oude Lansink, and I. J. M. de Boer. 2016. Environmental and economic performance of beef farming systems with different feeding strategies in southern Brazil. Agric. Syst. 146:70–79. doi:10.1016/j.agsy.2016.04.003

[CIT0103] Pelletier, N., R. Pirog, and R. Rasmussen. 2010. Comparative life cycle environmental impacts of three beef production strategies in the Upper Midwestern United States. Agric. Syst. 103:380–389. doi:10.1016/j.agsy.2010.03.009

[CIT0104] Picasso, V. D., P. D. Modernel, G. Becoña, L. Salvo, L. Gutiérrez, and L. Astigarraga. 2014. Sustainability of meat production beyond carbon footprint: a synthesis of case studies from grazing systems in Uruguay. Meat Sci. 98:346–354. doi:10.1016/j.meatsci.2014.07.005 25048094

[CIT0105] Pignati, W., N. P. Oliveira, and A. Silva. 2014. Vigilância aos agrotóxicos: quantificação do uso e previsão de impactos na saúde-trabalho-ambiente para os municípios brasileiros. Cien. Saude Colet. 19:4669–4678. doi:10.1590/1413-812320141912.12762014 25388175

[CIT0106] Platter, W. J., J. D. Tatum, K. E. Belk, J. A. Scanga, and G. C. Smith. 2003. Effects of repetitive use of hormonal implants on beef carcass quality, tenderness, and consumer ratings of beef palatability1,2. J. Anim. Sci. 81(4):984–996. doi:10.2527/2003.814984x 12723088

[CIT0107] Pollak, H . 2020. Pesticide footprint of Brazilian soybeans. A temporal study of pesticide use and impacts in the Brazilian soybean cultivation. Gothenburg, Sweden: Chalmers University of Technology.

[CIT0108] Ponnampalam, E. N., A. E. D. Bekhit, H. Bruce, N. D. Scollan, V. Muchenje, P. Silva, and J. L. Jacobs. 2019. Chapter 2 - production strategies and processing systems of meat: current status and future outlook for innovation – a global perspective. In: C. M. Galanakis, editor. Sustainable meat production and processing. Cambridge, Massachussets, USA: Academic Press; p. 17–44.

[CIT0109] Pryor, S. W., J. Smithers, P. Lyne, and R. van Antwerpen. 2017. Impact of agricultural practices on energy use and greenhouse gas emissions for South African sugarcane production. J. Clean. Prod. 141:137–145. doi:10.1016/j.jclepro.2016.09.069

[CIT0110] Rodrigues Paulino, P. V. R., M. A. Fonseca, L. Toledo Henriques, S. de Campos Valadares Filho, and E. Detmann. 2010. Nutritional requirements of Nellore cows and calves. In: S. de Campos Valadares Filho, M. I. Marcondes, M. L. Chizzotti and P. V. Rodrigues Paulino, editors. Nutrient requirements of Zebu beef cattle. Vicosa, Brazil: Federal Univeristy of Vicosa, Department of Animal Science.

[CIT0111] Rotz, C. A., B. J. Isenberg, K. R. Stackhouse-Lawson, and E. J. Pollak. 2013. Environmental footprints of beef production at the U.S. Meat Animal Research Center. The ADSA-ASAS Joint Annual Meeting, Indianapolis, IN, USA. 10.2527/jas.2013-6506 24146148

[CIT0112] Ruviaro, C. F., J. S. da Costa, T. J. Florindo, W. Rodrigues, G. I. B. de Medeiros, and P. S. Vasconcelos. 2016. Economic and environmental feasibility of beef production in different feed management systems in the Pampa biome, southern Brazil. Ecol. Ind. 60:930–939. doi:10.1016/j.ecolind.2015.08.042

[CIT0113] Ruviaro, C. F., C. M. de Léis, V. d. N. Lampert, J. O. J. Barcellos, and H. Dewes. 2015. Carbon footprint in different beef production systems on a southern Brazilian farm: a case study. J. Clean. Prod. 96:435–443. doi:10.1016/j.jclepro.2014.01.037

[CIT0114] Sawan, Z. M . 2016. Cottonseed yield and its quality as affected by mineral nutrients and plant growth retardants. Cogent Biol. 2(1):1245938. doi:10.1080/23312025.2016.1245938

[CIT0115] Smith, Z. K., P. T. Anderson, and B. J. Johnson. 2020. Finishing cattle in all-natural and conventional production systems. Open J. Anim. Sci. 10(2):17. doi:10.4236/ojas.2020.102013

[CIT0116] Smith, Z. K., and B. J. Johnson. 2020. Mechanisms of steroidal implants to improve beef cattle growth: a review. J. Appl. Anim. Res. 48(1):133–141. doi:10.1080/09712119.2020.1751642

[CIT0117] Stabile, M. C. C., A. L. Guimarães, D. S. Silva, V. Ribeiro, M. N. Macedo, M. T. Coe, E. Pinto, P. Moutinho, and A. Alencar. 2020. Solving Brazil’s land use puzzle: increasing production and slowing Amazon deforestation. Land Use Policy 91:104362. doi:10.1016/j.landusepol.2019.104362

[CIT0118] Stackhouse-Lawson, K. R., M. S. Calvo, S. E. Place, T. L. Armitage, Y. Pan, Y. Zhao, and F. M. Mitloehner. 2013. Growth promoting technologies reduce greenhouse gas, alcohol, and ammonia emissions from feedlot cattle. J. Anim. Sci. 91:5438–5447. doi:10.2527/jas.2011-4885. 24085413

[CIT0119] Stackhouse-Lawson, K. R., C. A. Rotz, J. W. Oltjen, and F. M. Mitloehner. 2012. Growth promoting technologies reduce the carbon footprint, ammonia emissions, and costs of California beef production system. J. Anim. Sci. 90:4656–4665. 22952364 10.2527/jas.2011-4654

[CIT0120] Statistica . 2021. Exports of beef and veal from Brazil in 2019, by destination (in million U.S. dollars). Accessed September 6, 2021. https://www.statista.com/statistics/617492/beef-and-veal-export-value-brazil-by-country-of-destination/

[CIT0121] Strydom, P. E . 2016. Performance-enhancing technologies of beef production. Anim. Front. 6(4):22–30. doi:10.2527/af.2016-0040

[CIT0122] Thompson, J. M., B. M. McIntyre, G. D. Tudor, D. W. Pethick, R. Polkinghorne, and R. Watson. 2008a. Effects of hormonal growth promotants (HGP) on growth, carcass characteristics, the palatability of different muscles in the beef carcass and their interaction with aging. Aust. J. Exp. Agric. 48(11):1405–1414.

[CIT0123] Thompson, J. M., R. Polkinghorne, M. Porter, H. M. Burrow, R. A. Hunter, G. J. McCrabb, and R. Watson. 2008b. Effect of repeated implants of oestradiol-17β on beef palatability in Brahman and Braham cross steers finished to different market end points. Aust. J. Exp. Agric. 48(11):1434–1441.

[CIT0124] United Nations . 2015. Sustainable development goals. Accessed November 8, 2017. http://www.un.org/sustainabledevelopment/sustainable-development-goals/

[CIT0125] United Nations . 2019. Growing at a slower pace, world population is expected to reach 9.7 billion in 2050 and could peak at nearly 11 billion around 2100. Accessed February 5, 2021. https://www.un.org/development/desa/en/news/population/world-population-prospects-2019.html

[CIT0126] USDA . 2019. Brazil - livestock and products – 2019 annual livestock report USDA - GAIN - global agricultural information network, Washington DC, USA.

[CIT0127] Vale, P., H. Gibbs, R. Vale, M. Christie, E. Florence, J. Munger, and D. Sabaini. 2019. The expansion of intensive beef farming to the Brazilian Amazon. Glob. Environ. Change 57:101922. doi:10.1016/j.gloenvcha.2019.05.006

[CIT0128] Valente, E. E. L., M. L. Chizzotti, C. V. R. Oliveira, M. C. Galvao, S. S. Domingues, A. C. Rodrigues, and M. M. Ladeira. 2015. Intake, physiological parameters and behavior of Angus and Nellore bulls subjected to heat stress. Semin. Cienc. Agrar. 36:4565–4574. doi:10.5433/1679-0359.2015v36n6Sup2p4565

[CIT0129] Vasconcelos, K., M. Farinha, L. Bernardo, V. do N. Lampert, M. Gianezini, J. S. da Costa, A. S. Filho, T. C. M. Genro, and C. F. Ruviaro. 2018. Livestock-derived greenhouse gas emissions in a diversified grazing system in the endangered Pampa biome, Southern Brazil. Land Use Policy 75:442–448. doi:10.1016/j.landusepol.2018.03.056

[CIT0130] Waclawovsky, A. J., P. M. Sato, C. G. Lembke, P. H. Moore, and G. M. Souza. 2010. Sugarcane for bioenergy production: an assessment of yield and regulation of sucrose content. Plant Biotechnol. J. 8:263–276. doi:10.1111/j.1467-7652.2009.00491.x 20388126

[CIT0131] Webb, M. J . 2018. Influence of production system on animal performance, carcass characteristics, meat quality, environmental impacts, production economics, and consumer preference for beef. Brookings, SD, USA: South Dakota State University.

[CIT0132] Webb, M. J., D. L. Pendell, A. A. Harty, R. R. Salverson, C. A. Rotz, K. R. Underwood, K. C. Olson, and A. D. Blair. 2017. Influence of growth promoting technologies on animal performance, production economics, environmental impacts and carcass characteristics of beef. Meat Muscle Biol. 1(3):23–24. doi:10.221751/rmc2017.022

[CIT0133] West, T. O., and G. Marland. 2002. A synthesis of carbon sequestration, carbon emissions, and net carbon flux in agriculture: comparing tillage practices in the United States. Agric. Ecosyst. Environ. 91:217–232.

[CIT0134] White, R. R., and J. L. Capper. 2014. An environmental, economic and social assessment of improving cattle finishing weight or average daily gain within United States beef production. J. Anim. Sci. 91:5801–5812. 10.2527/jas.2013-6632 24146151

[CIT0135] Wiedemann, S. G., B. K. Henry, E. J. McGahan, T. Grant, C. M. Murphy, and G. Niethe. 2015. Resource use and greenhouse gas intensity of Australian beef production: 1981–2010. Agric. Syst. 133:109–118. doi:10.1016/j.agsy.2014.11.002

[CIT0136] Yin, R. K . 1994. Discovering the future of the case study method in evaluation research. Eval. Pract. 15(3):283–290. doi:10.1016/0886-1633(94)90023-X

[CIT0137] Zu Ermgassen, E. K. H. J., J. Godar, M. J. Lathuillière, P. Löfgren, T. Gardner, A. Vasconcelos, and P. Meyfroidt. 2020. The origin, supply chain, and deforestation risk of Brazil’s beef exports. Proc. Natl. Acad. Sci. U. S. A. 117:31770–31779. doi:10.1073/pnas.2003270117. 33262283 PMC7749302

