# Supplementary material for: Modeling the effects of steroid implant use on the environmental and economic sustainability of Brazilian beef production
Source: Transl Anim Sci. 2021 Sep 20;5(4):txab144. doi: 10.1093/tas/txab144 (PMC8494015; doi:10.1093/tas/txab144)
Supplement: txab144_suppl_Supplementary_Materials [file txab144_suppl_supplementary_materials.docx]

**Table A1. Economic effects of implant use at low, medium and high levels of performance enhancement compared to no implants in Brazilian beef systems – revenue, costs, margin, return on investment and productivity per hectare of land**

|  | **No implants (NI)** | | **Low (LI)** | | **Medium (MI)** | | **High (HI)** | |
| --- | --- | --- | --- | --- | --- | --- | --- | --- |
| **Mato Grosso** | **Cow-calf** | **Finishing** | **Cow-calf** | **Finishing** | **Cow-calf** | **Finishing** | **Cow-calf** | **Finishing** |
| Revenue^1^ | 127 | 287 | 131 | 333 | 133 | 352 | 136 | 373 |
| Cash cost^1^ | 36.4 | 186 | 37.4 | 190 | 37.6 | 190 | 37.6 | 190 |
| Cash cost + depreciation^1^ | 56.1 | 200 | 57.1 | 204 | 57.2 | 205 | 57.3 | 204 |
| Gross margin^1^ | 90.1 | 101 | 93.7 | 143 | 95.5 | 162 | 98.1 | 183 |
| Net margin^1^ | 70.5 | 86.5 | 74.0 | 128 | 75.9 | 148 | 78.4 | 169 |
| Return on real investment^2^ | 3.48 | 1.54 | 3.51 | 1.75 | 3.54 | 1.85 | 3.61 | 1.96 |
| Return on real investment^3^ | 2.26 | 1.43 | 2.30 | 1.63 | 2.33 | 1.72 | 2.37 | 1.83 |
| **Mato Grosso do Sul** | **Cow-calf** | **Finishing** | **Cow-calf** | **Finishing** | **Cow-calf** | **Finishing** | **Cow-calf** | **Finishing** |
| Revenue^1^ | 129 | 224 | 129 | 249 | 130 | 260 | 131 | 271 |
| Cash cost^1^ | 35.9 | 190 | 36.4 | 194 | 36.3 | 194 | 36.3 | 195 |
| Cash cost + depreciation^1^ | 64.9 | 232 | 65.5 | 235 | 65.3 | 236 | 65.3 | 236 |
| Gross margin^1^ | 91.7 | 33.5 | 93.0 | 55.3 | 93.6 | 66.0 | 94.5 | 76.0 |
| Net margin^1^ | 62.7 | -7.69 | 64.0 | 14.09 | 64.6 | 24.8 | 65.5 | 34.8 |
| Return on real investment^2^ | 3.56 | 1.18 | 3.55 | 1.28 | 3.58 | 1.34 | 3.60 | 1.39 |
| Return on real investment^3^ | 1.97 | 0.97 | 1.98 | 1.06 | 1.99 | 1.11 | 2.00 | 1.15 |
| **Goias** | **Feedlot** | | **Feedlot** | | **Feedlot** | | **Feedlot** | |
| Revenue^1^ | 16,506 | | 19,111 | | 20,269 | | 21,446 | |
| Cash cost^1^ | 16,573 | | 16,361 | | 16,259 | | 16,157 | |
| Cash cost + depreciation^1^ | 16,939 | | 16,727 | | 16,625 | | 16,524 | |
| Gross margin^1^ | -66.9 | | 2,750 | | 4,010 | | 5,299 | |
| Net margin^1^ | -433 | | 2,384 | | 3,644 | | 4,922 | |
| Return on real investment^2^ | 1.00 | | 1.17 | | 1.25 | | 1.33 | |
| Return on real investment^3^ | 0.97 | | 1.14 | | 1.22 | | 1.30 | |

^1^ US$ per ha

^2^ Revenue/cash cost

^3^ Revenue/(cash cost + depreciation)
